# Characteristics of Effective Interventions Promoting Healthy Eating for Pre-Schoolers in Childcare Settings: An Umbrella Review

**DOI:** 10.3390/nu10030293

**Published:** 2018-03-01

**Authors:** Louisa Matwiejczyk, Kaye Mehta, Jane Scott, Emma Tonkin, John Coveney

**Affiliations:** 1 College Nursing and Health Sciences, Flinders University, Adelaide, SA 5000, Australia; kaye.mehta@flinders.edu.au (K.M.); emma.tonkin@flinders.edu.au (E.T.); john.coveney@flinders.edu.au (J.C.); 2 School of Public Health, Curtin University, Perth, WA 6845, Australia; jane.scott@curtin.edu.au

**Keywords:** dietary intake, healthy diet, pre-schooler, obesity prevention, social-ecological model, review

## Abstract

Early Childhood Education and Care (ECEC) settings have a pivotal role in shaping children’s dietary food habits by providing the contextual environment within which they develop these behaviours. This study examines systematic reviews for (1) the effectiveness of interventions to promote healthy eating in children aged 2–5 years attending centre-based childcare; (2) intervention characteristics which are associated with promoting healthy eating and; (3) recommendations for child-health policies and practices. An Umbrella review of systematic reviews was undertaken using a standardized search strategy in ten databases. Twelve systematic reviews were examined using validated critical appraisal and data extraction tools. Children’s dietary food intake and food choices were significantly influenced. Interventions to prevent obesity did not significantly change children’s anthropometric measures or had mixed results. Evidence was more convincing if interventions were multi-component, addressed physical activity and diet, targeted individual-level and environmental-level determinants and engaged parents. Positive outcomes were mostly facilitated by researchers/external experts and these results were not replicated when implemented in centres by ECEC providers without this support. The translation of expert-led interventions into practice warrants further exploration of implementation drivers and barriers. Based on the evidence reviewed, recommendations are made to inform child-health directed practices and policies.

## 1. Introduction

Good nutrition in early childhood is essential to ensure children reach their growth and developmental potential [1]. Furthermore, dietary health behaviours and food preferences are learnt early and carry through into adulthood [2,3]. In all ages, and increasingly in younger populations, poor food choices and overconsumption are associated with a higher risk of developing obesity [4,5]. Excessive weight developed in early childhood is particularly problematic as it is associated with an increased risk of developing physical, social and psychological conditions and earlier onset of non-communicable diseases (NCD) [6,7,8]. Contrary to popular belief, many children carrying extra weight do not outgrow it [9] and childhood-onset obesity is particularly difficult to address in later life [10]. As such, concern for children’s health, and escalating rates of NCD, have prompted the prioritization of healthy diets for young children globally [11,12].

Considerable public health effort and research have been directed towards nutrition interventions in the home and the school setting [13,14]. However societal changes to mothers’ workforce participation have increased the relevance of the childcare setting as a location for intervention in countries such as the United States, Canada, Europe, the UK and Australia. In the United States, more than 21 million preschool-aged children receive childcare and nearly 60% of these children receive centre-based childcare [15]. In Australia, nearly half of children under five years of age attend childcare with nearly a quarter receiving formal childcare [16]. Although hours vary considerably [17] children in many European Organisation for Economic Co-operation and Development (OECD) countries and 70% of Canadian pre-schoolers with working parents spend more than 30 h per week in formal childcare [18,19], where children receive up to 70% of their daily nutrition [20]. 

Although the home is still the primary influence [21,22], centre-based childcare has a pivotal role in shaping children’s dietary habits by providing a contextual environment within which they develop these behaviours. As such, experts have recommended that interventions promoting healthy eating and preventing obesity be targeted at childcare services [23,24]. In response to the plethora of research evidence this decade, several systematic reviews have been undertaken to investigate the effectiveness of lifestyle-related interventions in childcare. The systematic reviews relating to pre-schoolers and healthy eating have been predominantly about preventing or managing obesity, with a focus on the effectiveness of interventions which change weight status [25]. Other systematic reviews have focused on specific determinants of obesity such as diet, physical activity and other obesogenic behaviours including sedentary behaviour and sleep [26,27] or type of intervention such as educational and lifestyle interventions [28], influence of the food environment [29] or nutrition policies at child-care centres and impact on role modelling [30]. The range of research questions in these reviews has been wide, as have been the recommendations for decision-makers, practitioners and policy-makers. Given this surfeit of systematic reviews, a review is warranted of existing reviews to provide a concise overall examination of the large and diverse body of information. 

Umbrella reviews are becoming relatively common [31,32] as a means of providing an overall examination of a broad range of topics within a similar area of interest [33,34]. A growing number of guidelines and resources address the methodological rigour of this type of evidence synthesis [33,35,36]. Umbrella reviews only use the highest level of evidence, that is, other systematic reviews, and provide a means to compare and contrast the findings from different systematic reviews as well as a summary of the evidence for healthcare decision-makers [33]. This is the first Umbrella review to provide a systematic examination and overview of a broad range and number of reviews investigating the effectiveness of interventions and practices promoting healthy eating behaviours in 2–5 years old in centre-based childcare. 

### Objectives

The primary aims of this Umbrella review are to examine previously published systematic reviews to determine (1) the effectiveness of interventions to promote healthy eating in children aged 2–5 years attending centre-based childcare; (2) intervention characteristics which are associated with successfully promoting healthy eating in pre-schoolers; and (3) recommendations for child-health directed policies and practices.

## 2. Materials and Methods

### 2.1. Search Strategy and Eligibility Criteria

To identify possible systematic reviews the online bibliographic databases Medline, Emcare (New York, NY, USA), PsycINFO (Washington, DC, USA), Embase (Amsterdam, Netherlands), CINAHL (Ipswich, MA, USA), Health Technology Assessment Database, ERIC, Scopus, Web of Science Core Collection, Joanna Briggs Institute (JBI) Evidence-Based Practice Database of Systematic Reviews and Cochrane Database of Systematic Reviews were searched for reviews published between January 2000 and September 2017. The search strategy is available as Table S1: Record of search strategies in an online repository of supporting materials. In addition to the online search, relevant grey literature sources were searched including key government and organisational websites, National Library catalogues, conference proceedings, theses repositories, and clinical trial registries. The literature search of reviews not produced by commercial publishers was restricted to reports produced since January 2000 from comparable high-income countries, including Australia, New Zealand, Canada, Europe, the United Kingdom, and the United States [37]. The JBI Database of Systematic Reviews and Implementation Reports, the Cochrane Database of Systematic Reviews, and the PROSPERO register were searched for prospective systematic review protocols. Reference lists of included systematic reviews were checked to identify any missed studies. Reviews were those published post-January 2000, as few systematic reviews, in general, were published using Preferred Reporting Items for Systematic Reviews (PRISMA) prior [33] and most primary studies relating to lifestyle and childcare have been published in the last decade [38,39]. No language limitations were applied. Reviews were included if they met the PICO-derived inclusion criteria: (1) reviews of studies of children aged 2–5 years attending centre-based childcare (defined as regulated childcare held outside of the home and provided by non-relatives, also known as nurseries, day care, preschools, long day care and kindergarten) or of childcare educators (those directly working with children and those indirectly working with children including cooks); (2) reviews of studies which considered interventions or behaviour change strategies with the intent to improve or promote healthy eating; (3) reviews of studies of any study design, with or without a comparison group, with outcomes measured at baseline and post-intervention; (4) reviews of studies with measurable outcomes for food and dietary behaviours or nutrition practices. Reviews considered included systematic reviews, meta-analysis, overviews of reviews, review of reviews and narrative reviews. 

The following reviews were excluded: (1) studies with infants or studies where the children were attending compulsory schooling usually six years or older; (2) studies treating children for obesity or a clinical related condition; (3) studies using school, the home or settings which are not registered childcare; (4) studies in which dietary behaviour or dietary-related outcomes were secondary outcomes and not separately reported; (5) studies focused on low-income countries. Although the search strategy did not limit studies to particular countries, only systematic reviews relating to high-income countries as defined by the OECD (2017) were included because the childcare arrangements and practices are similar. An a priori protocol for the Umbrella review was registered with PROSPERO (CRD42017078749). 

### 2.2. Assessment of Methodological Quality and Data Extraction

To assess the methodological quality of the reviews and to determine the extent to which reviews had addressed the possibility of bias in the design, conduct and analysis, the Johanna Briggs Institute (JBI) Critical Appraisal Checklist for Systematic Reviews and Research Syntheses was used [33]. This validated 11-item checklist has been subjected to extensive peer review. Two reviewers independently (L.M., E.T.) assessed the eligible reviews after discussing each item in the appraisal instrument to gain a common understanding of what constitutes appropriate levels of information and the criteria for a positive, negative or unclear response. After the independent assessment, the two reviewers met to discuss the individual items for each study and if there was disagreement, a third reviewer independently reviewed the study to resolve the decision (J.C.).

To guide the extraction and synthesis of data from the selected studies and minimize the risk of author bias, a standardized tool, the JBI Data Extraction Form for Systematic Reviews and Research Synthesis [40] was employed independently by the same two reviewers. Information extracted from each review included the following: (1) Review characteristics: author/year, objectives, participants (characteristics/total number), setting/context, interventions of interest, number of databases/sources searched, date range of included studies, number of total studies included, detailed description of the included primary studies related to healthy eating promotion (number/type of studies/country of origin), appraisal instrument and rating, method of analysis and outcomes assessed; and (2) Review Results: significance/direction, heterogeneity and significant findings/outcomes of the review. Prior to the process, the two reviewers discussed each of the tool’s items for a common understanding and to identify any additional data which might need to be extracted. It was agreed to also include: factors or characteristics of interventions that influence intervention effect, the use of any underpinning behaviour change or health promotion theories, author recommendations for practice and author recommendations for research. Following this process and discussion, if there was any uncertainty with data extraction, a third experienced reviewer was consulted (J.C.).

## 3. Results

### 3.1. Study Selection Process

The study selection process is summarised in Figure S1: PRISMA flowchart of the selection process for systematic reviews (available in the online data repository). In total, 1785 citations were initially identified. After duplicates were removed the title and abstract of 983 citations were screened for relevance and 21 studies were identified for full-text analysis. Four additional studies were included from manual searching of references and citation snowballing [41,42,43,44]. A search of the grey literature did not identify any additional eligible reviews. The 25 full-text systematic reviews were screened and 11 systematic reviews excluded [14,42,43,44,45,46,47,48,49,50,51] because the dietary outcomes were not separately reported, there was too little information, the age group related to children attending school and/or relevant outcomes were not measured (Figure S1). Fourteen systematic reviews were considered eligible for the present Umbrella review. Of the 14 included reviews, seven stated obesity-related physiological outcomes, for example, Body Mass Index (BMI) as the primary outcome, with diet-related outcomes reported separately as secondary outcomes [38,39,52,53,54,55,56]. The other seven studies, addressed diet-related behaviours as the primary outcomes [23,41,57,58,59,60,61]. Two systematic reviews were excluded when assessed for methodological quality [23,41]. The methods of these reviews were not described in enough detail to determine robustness and were published before PRISMA guidelines were used. Agreement between the two reviewers was strong and statistically significant (Kappa score *p* < 0.0005).

### 3.2. Description of Reviews

Twelve systematic reviews were included in the final review and the quality assessment ratings are tabulated in Table S2: Critical appraisal results for the included reviews in the Supplementary Materials. The reviews met the 11-item validated JBI quality assessment criteria, except for five reviews where one or two criterion was not met or unclear, but these anomalies were judged not to warrant exclusion. In two reviews it was unclear if both the process of appraisal and data extraction was undertaken independently by two reviewers. In Sisson et al. (2016) criteria for appraising the studies were on purpose not included to ensure a broad inclusion of studies. In Hesketh & Campbell (2010) limitations for search selection was not justified but the included studies were consistent with other reviews. In Nixon et al. (2012) methods to minimize errors in data extraction were not reported.

Table 1 provides an overview of selected characteristics of the included reviews. Reviews included primary studies all post-2000 apart from six primary studies examined by Ward, Bélanger et al. (2015) and Ward, Welker et al. (2016). The total number of included primary studies which were unique was 101 and ranged from three [38] to 45 [54]. A relatively small number of primary studies were excluded by the reviewers (Table S3: Characteristics of included systematic reviews). Reasons for ineligibility of some of the primary studies were no dietary outcomes reported or settings such as schools and Family Day Care [39,53,57]. The total sample size of the studies included in the individual reviews ranged between 260 children [60] to more than 18,000 [53,57] and centres caring for between six [60] and more than 1050 children [61]. The majority of the primary studies were conducted in the USA with smaller numbers in other high-income countries including Australia, Israel, Europe (Switzerland, Germany, Belgium, France, Netherlands and Spain), UK, Asia and South America. Three primary studies were undertaken in high-middle income countries, China [62], Turkey [63] and Columbia [64].

Most of the primary studies in all of the reviews were randomised control trials (RCT) or cluster-RCT followed by case-control trials or quasi-experimental studies (Table 1). Concerns about the quality of the evidence were raised in all of the reviews, particularly where dietary changes were the primary outcomes [57,58,61]. Based on the data reported in the 12 reviews, more than half rated at least 50% of the primary studies as weak [55,57,59,60] or having insufficient information to permit evaluation [52,56,61]. Three studies using Cochrane tools did not allocate a quality rating as there was a high-risk bias for at least one domain [38,56,61]. Only the review by Mikkelson et al. (2015) rated 22 of the 26 primary studies as having a moderate or strong quality of evidence. The other four reviews rated the majority of the studies as moderate [38,39,53,54]. Implications are that results are uncertain and must be considered with caution. However, most of the studies were RCT or cluster RCT, which is a high level of evidence, and reviews were selected using rigorous quality assessment. Nevertheless, sample sizes of less than 30 centres, most of the studies being from the USA and studies with a high risk of bias because they were not RCT, may limit the generalisability of the results. 

There was considerable heterogeneity between primary studies which precluded pooling of the data and meta-analysis or any systematic reviews undertaking Grading of Recommendations Assessment, Development and Evaluation (GRADE) [65]. Heterogeneity existed in studies’ objectives (e.g., obesity-related physiological objectives, dietary-related objectives), how dietary-related outcomes were measured (e.g., self-reported dietary intake, 24 h-recall, plate wastage measurements) and level of intervention (e.g., individual-level with a focus on knowledge, attitude, beliefs; environmental-level with a focus on changes to food provision and policy, socio-cultural elements or both). 

### 3.3. Findings of the Reviews

#### 3.3.1. Effectiveness

##### Dietary Intake

Study findings favoured dietary effectiveness in most of the included reviews (Table 1). Assessed outcomes were all in the direction of nutritional improvement when measured for children’s dietary intake and food choices. For those studies seeking to improve children’s eating habits, significant improvements in children’s dietary intake was reported in eight reviews and included an increased intake in children’s mean servings of fruit and or vegetables [56,57,58] as well as decreased intake of total fat and saturated fat [38,57,58]. Moreover, most reviews which included interventions which influenced centre food provision or parental provision of lunchboxes reported post-intervention improvements in the number and mean size servings of fruit and/or vegetable offered to children [56,57], fewer sweetened beverages [57] and fewer energy dense and nutrient poor (EDNP) foods [56,57]. No intervention improved the implementation of all policies and practices recommended to strengthen healthy eating environments and educator behaviours relative to a comparison group [61] but most reviews reported that primary studies had achieved a significant change in at least one measured variable specific to food groups such as fruit, vegetables or nutrients [54,55,61]. 

##### Weight Status

Seven reviews focused on obesity-prevention and obesogenic behaviours (including diet-related behaviours). Despite reporting significant effects on BMI and other measures of adiposity for some primary studies, review authors concluded overall that diet-related interventions did not have a consistently positive impact (Hesketh and Campbell 2010, Nixon, Moore et al., 2012, Morris, Skouteris et al., 2014, Zhou, Emerson et al., 2014, Ling, Robbins et al., 2016, Sisson, Krampe et al., 2016, Ward, Welker et al., 2016). Two other reviews reported no significant changes in weight status (Mikkelsen, Husby et al., 2014, Wolfenden, Jones et al., 2016). Ling, Robbins et al. (2016) and Zhou, Emerson et al. (2014) reported that the primary studies which significantly affected weight outcomes were multi-component interventions which addressed both dietary and physical activity behaviours. Mikkelsen, Husby et al. (2014) reported that single component interventions did not have a significant effect on children’s fruit and vegetable intake but five of six multi-component interventions did. Actively involved and engaged parents were also associated with consistently positive impacts on children’s weight status (Nixon, Moore et al., 2012, Sisson, Krampe et al., 2016).

##### Multi-Level Interventions

More positive outcomes were seen in reviews assessing interventions directed at both environmental- and individual-level determinants of healthy eating behaviours. Most effective were multi-level interventions targeting environmental-level determinants including implementation support [54,55,58,61]. Several reviews reported improvements in educators’ nutrition knowledge and diet-related practices in intervention groups [57,58]. Interventions which focused on educators’ practices at mealtime and children’s eating behaviours also resulted in significant outcomes [59]. Similarly, children significantly influenced other pre-schoolers’ food choices and food preferences through role modelling and observational learning, particularly with fruit and vegetables [60]. Children’s knowledge also improved significantly following educational activities [53,57,58].

Three reviews reported that the strongest effects came from interventions targeting environmental-level determinants. Bell and Golley (2015) examined 13 primary studies, with 12 reporting significant improvement in the food provided in centres (through food policy and changes in educators’ practices), the nutritional quality of menus and parental food provision of lunchboxes. Primary studies on interventions focusing on environmental-level factors reported positive outcomes including food and nutrition policies and the food environment, however few of these studies also reported on whether children’s dietary intake had changed as a result [54]. Wolfenden, Jones et al. (2016) reported that interventions targeting the food environment were most successful but did not have a significant effect on other outcomes such as a child’s diet or weight status. 

##### Parental Involvement and Engagement

Half of the reviews reported an association between parental involvement and engagement, and achievement of objectives in ECEC interventions [38,52,53,54,55,58]. The classification of parental involvement as none, low or passive, moderate or active or high was different across the reviews. Parental involvement was typically classified as active if parents were involved in a component of the intervention, for example, an education program or hands-on experiences [39]. Intervention effects on children’s anthropometry were weak and inconsistent but improved when involvement and engagement with parents occurred [53,54,55]. Using a custom-designed intervention intensity coding system, Ward, Welker et al. (2016) found that interventions with any parental engagement component significantly added to the effectiveness of the ECEC intervention. Morris, Skouteris et al. (2014) found positive weight changes in six primary studies and improvements in healthy eating in most studies (*n* = 15). Six primary studies attributed high parental engagement to the successful achievement of their primary outcome to effect changes in children’s weight (cited in Morris, Skouteris et al., 2014).

### 3.4. Characteristics of Successful Interventions

#### 3.4.1. Delivery of Interventions

Positive outcomes for healthy eating behaviours were mostly reported for interventions delivered by researchers or external experts [39,52,55,56]. All of the included primary studies in the review by Wolfenden, Jones et al. (2016) were externally-delivered by nurses, health service personnel, dietitians or other experts. A quarter of the primary studies were delivered by childcare educators in the review by Ward, Welker et al. (2016) and although there were fewer positive dietary-related outcomes there was no difference when anthropometric outcomes were compared with strategies delivered by external researchers. The most commonly used implementation strategies were staff group education and training sessions, written materials, the inclusion of nutrition-related activities in the childcare curriculum and food and nutrition policies [57].

#### 3.4.2. Behavioural Change Theories

Eight reviews reported the number of included primary studies which used a theoretical framework [39,52,53,54,56,57,58,61] and are reported in Table S4: Summary of the evidence from selected reviews. The majority of reviews listed between a third and two-thirds of the primary studies as having a theoretical framework. The most common theoretical frameworks used were behavioural change theories (BCT) including the social ecological model (SEM) and social cognitive theory (SCT) or social learning theory (SLT). Theoretical frameworks used in fewer than two included primary studies were the: Health Belief Model, Social Determination Theory, Jajonc’s mere-exposure theory of effect, Piaget’s Developmental Theory, Multiple Intelligence Theory, a transtheoretical model for behavioural change and a capacity building model. Reviews which identified theoretical underpinnings found that most of the studies were developed without considering theoretical models or frameworks [52,56,58,61]. Nixon et al., 2012 and Sisson et al., 2016, who examined any associations with theory and outcomes, found that studies that used SCT/SLT when developing an intervention had significant favourable outcomes in one or more outcomes and that there were a greater number of effective studies which utilised behavioural theory frameworks. Sisson et al., 2016 noted that 25 of the 29 theory-based dietary-related interventions were effective, however, all 14 non-theory based interventions were also somewhat effective. 

#### 3.4.3. Characteristics of Interventions Involving Educators

Most of the dietary-related interventions targeted educators’ behaviours and practices and included nutrition education and training sessions [57]. Educational interventions changed educators’ knowledge [57,58], although Wolfenden, Jones et al. (2016) reported that knowledge was not significantly affected. Children’s acceptance and intake of health-promoting foods increased if educators modelled healthy eating enthusiastically [57,58,59,60], used immediate positive verbal reinforcement and served fruit and vegetables in advance of other foods [59]. Using non-food rewards, encouraging ‘try one more bite’ and allowing children to self-select food was also effective [59]. Workplace interventions supporting educators’ wellness and lifestyle also had promising results [54,57]. 

#### 3.4.4. Characteristics of Interventions Directly Involving Children

Effective interventions involving children included interactive educational activities as part of the childcare curriculum [39,52,54,57,58] and using children as role models [57,58,59,60]. Girls were more influential as role models for trying and consuming healthy foods for both genders and younger children were more influenced by watching older children as to what to eat [60]. Children also ate more in larger peer groups and tended to choose the same food as the previous child [60]. 

#### 3.4.5. Characteristics of Interventions Involving Parents

Active parental involvement included participation in any intervention component such as receiving written material, receiving regular newsletters, attending education sessions or workshops, completing homework tasks, participating in curriculum planning or participating in interactive hands-on activities such as cooking, growing vegetables or similar activities, with their children [38,39,52,53,54,58,60]. Even ‘low’ participation of parents such as receiving written material was associated with more positive outcomes [38,39,52,53,54,55]. 

### 3.5. Review Recommendations

Recommendations for practice and policy (Table 2) included: (1) underpinning intervention design with theoretical frameworks and effective behavioural change theory; (2) targeting intervention strategies at environmental-level and individual-level determinants with a multi-component, multi-level approach; (3) involving and engaging parents in intervention strategies; (4) building the capacity of educators, parents and of children. Successful training included goal setting and increased self-efficacy and self-regulation through feedback. Skill development was enhanced with role modelling and opportunities for observational learning.

Summarised recommendations for future research (Table 2) included: building upon existing activities, including cost-effectiveness assessment in the evaluation, being driven by user involvement (educators, parents) and children’s views, measuring children’s dietary changes as well as environmental impact and having longer follow-up. Meta-analysis is required, with more high-quality randomised control trial (RCT) with larger sample sizes using validated measurement methods and tools.

## 4. Discussion

This Umbrella review investigated the effectiveness of interventions to promote healthy eating in children aged 2–5 years attending centre-based childcare. The aim was to also identify characteristics of successful interventions and list and summarise the most frequent recommendations for policy, practice and research. Overall, 12 systematic reviews of acceptable methodological quality were included examining 101 primary studies.

### 4.1. Implications for Practice and Policy

**Figure 1:**
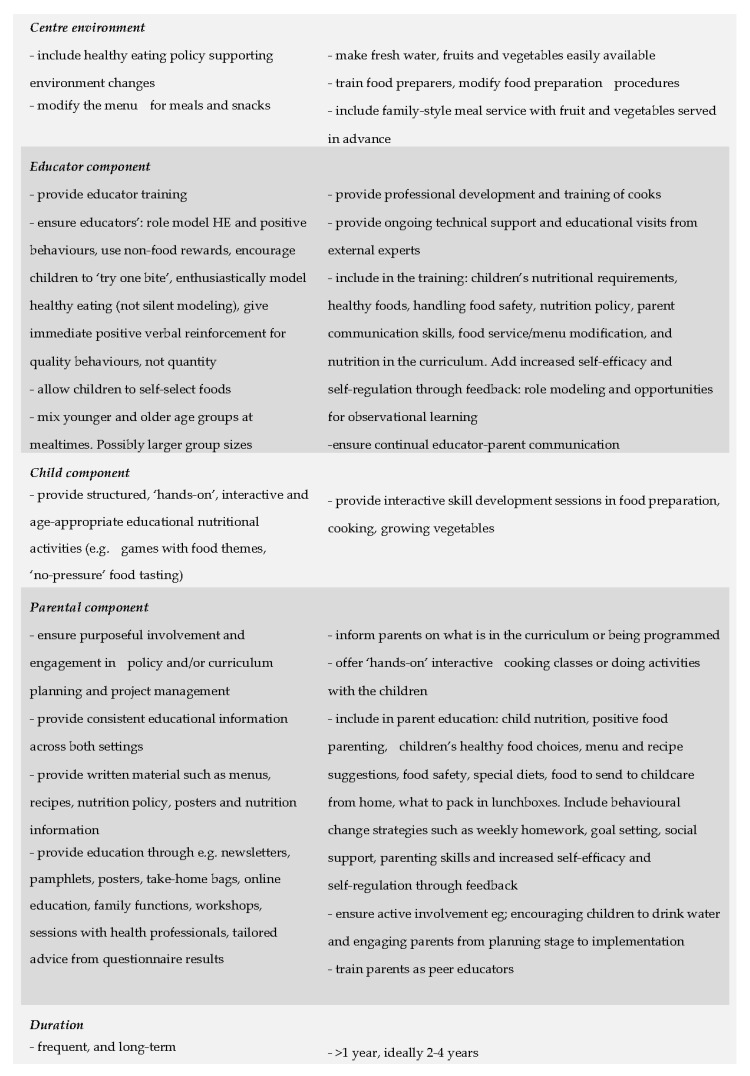
List of summarised multi-strategy, multi-level intervention characteristics.

Despite the considerable heterogeneity, the review findings supported the proposition that interventions to promote healthy eating in children aged 2–5 years attending centre-based childcare are effective. Successful interventions were multi-component, multi-level targeting both environmental and individual-level determinants of healthy eating behaviours. Multi-component interventions included educational strategies, changes to the centre-environment and policy. These findings are consistent with the conclusions of other Umbrella reviews for other settings [13,66,67,68] and public health priorities [69,70]. Overall, institutional changes facilitated by policies, age-appropriate health promoting curricula and educators’ training were recommended [56,61]. Involving educators as role models and interventionalists may improve children’s dietary food patterns, particularly if educators are given professional development, training and ongoing technical support [52,58,61]. The key characteristics associated with successful outcomes are summarised in the textbox (Figure 1: List of summarised intervention characteristics). 

A common recommendation in the reviews was to underpin intervention design with theoretical frameworks and effective behavioural change theories, ideally components of Social Cognitive Theory [71] alongside a social-ecological framework [72]. Wolfenden et al., 2016, suggest that if an intervention is developed using a comprehensive theoretical framework it would be more likely to be effective as it would address the theoretically identified barriers and facilitators. This aligns with the conclusions of most of the reviewers that multiple factors influence diet-related behaviours and require multiple strategies and levels of influence [73]. Consistent with the social-ecological model, interventions with the biggest impact focused on environmental changes such as menu modifications, policy and changes to food provision [38,57,61] coupled with technical support and training [54]. Multi-component approaches addressing the centre’s environment as well as the inclusion of an educational component were more effective than education alone [58] and are consistent with findings in other settings [28,66]. 

### 4.2. Evidence Gap

The translation of changes in educators’ knowledge, practices and centre environment to children’s dietary behaviours was however not consistently observed [39,54]. Moreover, positive changes in weight status to prevent obesity through dietary-related interventions reported in the reviews were not always achieved. Positive changes in weight status were attributed to interventions which addressed both diet and physical activity [52,55,58] and also actively involved and engaged parents [39,54]. 

More studies assessing the dietary-related outcomes from involving and engaging with parents are required. Even small levels of parental involvement were associated with better weight status outcomes [38,39,52,53,54,55]. Parents were however rarely fully engaged [58]. Being fully involved included parents knowing what children were learning, participating in curriculum planning, attending nutrition education sessions and participating with hands-on interactive educational activities, with or without their children [39,52]. In the one primary study that measured the impact of parental involvement on child diet-outcomes [74], parental satisfaction was correlated with children’s weight change. Parents who were satisfied with the program consumed fewer energy-dense nutrient-poor foods suggesting parental involvement and satisfaction could be linked with more effective outcomes. 

More research is needed to understand the interactions between educators and parents and the impact of collaborative parental engagement. With many children spending time in childcare and the premise that all food preferences are learnt, educators’ roles are crucial as very young children are dependent upon them not only to provide food but also to guide and shape their food preferences and dietary habits [75]. Qualitative studies have explored educators’ perception of the influencers on children’s diets [24,75,76,77] and identified the importance of parental involvement. Educators have a role in inviting parental participation and this, along with building the capacity of educators through technical support and training, was recommended by several reviewers [52,53,56,57,59]. Interventions are needed to build the confidence of educators to engage with and involve parents and extend key messages across the two settings. 

The impact of nutrition-related strategies to build the capacity of children is also an evidence gap. Findings emphasized the importance of targeting children with interactive education and hands-on experiences which are age-appropriate [39,52]. This is consistent with recent studies that these interventions influence children’s food preferences and readiness to try new foods [78]. Nixon et al. (2014) further recommended that the interventions should be informed by children’s knowledge and behaviours and the impact of this and age-appropriate education is a recommended area of emerging research. 

The impact of nutrition-related interventions and practices on children from low socio-economic areas is of particular interest. Many of the primary studies were directed at centres in low socio-economic areas or centres with a high proportion of children from disadvantaged families. The outcomes suggest that interventions supporting these populations could help reduce health inequalities [58]. This observation is similar to findings from diet-related studies in other low-income settings [79] and supports the call for focusing efforts in this area although results for pre-schoolers was modest but promising [79]. 

Missing from this Umbrella review was evidence of the sustainability of dietary-related interventions as few primary studies were implemented for more than a year and/or outcomes measured after the intervention. The recommended duration is at least one year, ideally 1–2 years [56,58]. Notable exceptions to this were the Head Start and Healthy Start programs in preschools for socioeconomically disadvantaged children in the United States [80,81,82,83,84,85,86] and a program of limited interventions in China, Germany, France, Belgium, Spain and Australia [62,87,88,89,90,91,92,93]. Interventions need to be of a duration with follow-ups that allow enough time for changes to take effect [56,58]. This is a gap for future research as is the impact of the comprehensiveness (intervention complexity) of interventions. 

Although the more comprehensive the intervention, the more likely it is to be successful, comprehensiveness may affect feasibility and fidelity negatively and warrants further exploration. Ward, Welker et al. (2016) found an inverse relationship between comprehensiveness and positive outcomes. Furthermore, in an Umbrella review investigating community-based interventions promoting healthy eating and physical activity, multi-component interventions were not correlated with positive outcomes [70]. Moreover, most of the primary studies in this Umbrella review were externally-delivered and the results not replicated when delivered by educators. It is not unusual for the effectiveness of interventions to be lost when it is adapted for the local context in the non-research setting [94]. The translation of knowledge and evidence-based recommendations into practice is a universal challenge for researchers, practitioners and policy-makers [95]. Formative and qualitative research is therefore needed to understand the local context, determine barriers to strategy implementation and focus on implementation drivers and barriers to increase understanding of how interventions work [56,58,61]. This would enable the involvement of the users (educators, parents) to more fully, incorporate children’s views and provide the engagement needed for more sustainable as well as effective outcomes.

Lastly, reviewers recommended that cost-effective studies be undertaken [38,56,61]. Lifestyle interventions are likely to be cost-effective for pre-schoolers [96] and childhood obesity is associated with excess healthcare expenditure [97].

### 4.3. Limitations of the Studies

Based on the data reported in the 12 reviews, reviewers cautioned that many primary studies were rated as weak or having insufficient information to permit evaluation. The actual effect of the intervention may therefore be smaller than the effects reported because of the low quality of reporting [98] and generalizing the results needs to be used cautiously. Although more RCT with larger samples sizes are called for, the nature of original studies in the real-world environment of ECEC settings, however, means that they are not feasible. A more pragmatic research approach is needed [99] focusing on existing activities. By combining quantitative and qualitative research into the same investigation, qualitative research can be used to confirm the quantitative findings and explore how evidence can be translated into practice more effectively [99]. If the primary studies were designed to be more homogenous, data could be pooled and examined using GRADE which does not categorize studies as weak because they are not RCT. 

### 4.4. Limitations and Strengths of the Umbrella Review

Some of the challenges identified by Pollock et al. (2017) and Ballard & Montgomery (2017) in their critique of the robustness of Umbrella reviews were encountered in this study. These challenges included primary studies overlapping between reviews and appearing in more than one review, and a mismatch between the scope of the systematic review being examined and the research question of the Umbrella review. Seven of the 12 reviews had a remit for obesity prevention rather than healthy eating as a primary outcome. Furthermore, the heterogeneity of the reviews and the assessment of insufficient information in three reviews precluded an evaluation of the quality of the research through the use of GRADE. This was compounded by the difficulty for the systematic reviews to apply GRADE or a meta-analysis for the same reason. To address these challenges, the primary studies which appeared in more than one review were identified and the extent of overlap considered. Moreover, in the data extraction stage, within these accepted reviews, only primary studies which met the scope of the Umbrella review were included, strengthening confidence in the findings. Mapping the overlapping primary studies reassured the authors that the search strategies were through and demonstrated consistency between reviewers. To further ensure methodological strength, the scope of the Umbrella review was limited to only those reviews where diet-related behaviours and measures were reported separately, PRISMA guided the search strategy and two validated tools were used to assess the quality and risk of bias of the reviews and to standardize data extraction [33]. The consistency of the findings and recommendations between the reviews supported the justification of this process. Similar to the findings by Pollock et al. (2017) this Umbrella review was able to identify evidence gaps and meet its objectives. 

## 5. Conclusions

Interventions promoting healthy eating positively influence children’s dietary food patterns. Although environmental-level and individual-level determinants of healthy eating are impacted by centre-based interventions, these effects are not consistently translated to changes in children’s diet-related behaviours or anthropometrics as a measure of preventing obesity. Positive outcomes can be further strengthened with parental involvement and engagement, and multi-level, multi-component strategies are recommended. Comprehensiveness may, however, affected feasibility and fidelity negatively when enacted by end-users; therefore studies on existing interventions implemented by end-users are recommended. Meta-analysis and stronger study designs are called for but are often not feasible in the real world of childcare. Therefore the translation of research or expert-led interventions into practice warrants further qualitative exploration of implementation drivers and barriers with end-users. This understanding and end-user involvement may contribute to the sustainability of interventions which is rarely reported.

The summarised findings and recommendations from this Umbrella review can inform child-health directed policies and practices. Based on the evidence, public health effort is warranted to support healthy eating interventions and practices in centre-based childcare. By incorporating multi-level and multi-component interventions into routine practices and extending this across the home and childcare setting, healthy food preferences and dietary-related behaviours can be influenced. More successful interventions require high levels of parental engagement, the use of behaviour change strategies and a focus on building the capacity of educators, children and parents.

## Figures and Tables

**Table 1:** Key Characteristics of the Selected Systematic Reviews.

Author/Date	Objectives Examined	Number of Primary Studies Included in Each Review/Total Number of Diet-Related Studies. Participant Characteristics	Study Design	Key Findings of the Review	Overall Recommendations of the Review
Bell and Golley 2015	Effectiveness of nutrition promotion interventions on children’s dietary intake.	24/25 Children 0–5 years, providers and staff or parents of children, formal childcare	Prospective studies with or without a comparison group, outcomes measured at baseline and post intervention 4 RCT, 1 cross-over cluster-RCT, 8 CCT, 10 cohort, 1 cross-over, 1 cross-over quasi-experimental	ECS interventions can achieve changes in children’s dietary intake and associated social-environmental determinants. DI; Significant effect on children’s dietary intake (8/11). Significant improvements in centres nutrition environment (6) including policy (2), nutrition best practices (3), nutritional quality of centres’ menus (3), parental food provision (4), child knowledge/attitudes/preferences (2), and staff knowledge/attitudes/behaviours (2).	ECS are potential settings for effective nutrition health promotion Environmental interventions can achieve dietary improvements Evaluate effect of nutrition environment changes on children’s dietary intake Utilise age-appropriate behaviour change theory
Hesketh and Campbell 2010	Effectiveness of interventions to prevent obesity, promote healthy eating and/or physical activity or reduce sedentary behaviours.	3/9 Children 2–5 years, preschool/formal childcare	Experimental studies 2 cluster-RCT, 1 CCT	Achieved success in modifying outcomes of interest. AN: Significantly lower BMI increases at 1 and 2 years follow up in one study. Two studies significant decrease in serum cholesterol but no change to height-weight ratio. DI: Significant decrease in saturated fat and total fat in snacks, and corresponding reduction in intake in two studies	Add parental component. Build knowledge and skills of educators and parents Consider SBT-based strategies Build on existing research activities Need cost-effective studies
Ling, Robbins et al., 2016	Effects of prevention and management interventions on overweight/obesity.	13/16 Children 2–5 years, formal childcare	Intervention studies with a sample > 30 centres 13 cluster-RCT	Studies which combined diet with PA, had a significant effect on measures of BMI (6/13). Findings supported teaching preschool children with interactive education and their families with interactive education and behavioural therapy. Lack of parental involvement may account for limited success in all studies.	Build knowledge and skill capability of educators with education, and health-promoting component for educators. Build HE capacity of both parents and children. Offer parents interactive education and nutrition-related behavioural therapy. Use age-appropriate interactive, hands-on experiences with children
Mikkelsen, Husby et al., 2014	Effectiveness of different strategies influencing children’s food choice at an early age.	26 studies Children 3–6 years, preschools/formal childcare	Intervention studies with baseline and follow-up measurements 11 RCT, 9 quasi RCT, 1 cross-over, 2 pre-post test design, 3 cluster-RCT	Comprehensive interventions more likely to succeed in behaviour change, especially when targeting children of low-income families. Multi-component programs which included education, changes to the centre environment, policy and involvement of parents were most effective. DI: Significant increase in fruit and vegetable intake and in nutrition knowledge in relevant studies. AN: No significant effect	More comprehensive interventions likely to be more successful i.e., multi-component and multi-level Target disadvantaged groups Add longer follow-up Focus on implementation drivers and barriers to increase understanding of what makes an intervention work
Morris, Skouteris et al., 2014	How have parents been incorporated into ECEC childhood obesity interventions and to what extent, if any does their involvement impact the outcomes of the intervention?	12/15 Parents of children in preschools/formal childcare	Experimental studies 2 RCT, 6 cluster-RCT, 3 quasi-experimental, 1 prospective cohort	AN: Positive and significant weight changes in some studies (6/12). No changes in anthropometry in all studies despite change in parental and child knowledge and attitudes and child unhealthy-diet behaviours. DI: Secondary outcome relating to healthy eating seen in most studies.	Build capacity of educators and parentsIncrease educators’ role in parental engagement Include collaborative parental involvement, including in curricula Future research on collaborative parental involvement and effects
Nixon, Moore et al., 2012	Identify effective behavioural models and behaviour change strategies, underpinning preschool and school-based interventions aimed at preventing obesity.	4/9 Children 4–6 years, pre-schools/formal childcare	Intervention studies with before and after measures in the same children plus follow-up of 6 months or longer 1 RCT, 3 cluster-RCT	Interventions that combined high levels of parental involvement, interactive learning plus targeted dietary change with long-term follow-up were most effective. DI: significant favourable changes in dietary behaviours (4/4). AN; significant favourable changes in intervention group (2/2).	Include BCS Build children’s (and parents) perceived competency to make dietary changes with education and modelling positive behaviours Change centre-environment and measure impact Ensure evidence-base driven by users involvement
Sisson, Krampe et al., 2016	Effectiveness and description of interventions that target obesogenic behaviours in child care centres.	45/71 Children 3–5 years, childcare settings	Experimental studies 22 RCT, 19 quasi-experimental or pre-post design, 3 natural experiments	DI: Most studies achieved a significant effect in at least one nutrition outcome (87% desired effect).	Multi-level (child, environment), multi-component Focus on childcare environment including technical support and training Include parental involvement Include BCS e.g., SEM, SCT Focus future research on RCT underpinned with BCT with emphasis on parental involvement Measure environmental effects on child’s dietary intake
Ward, Welker et al., 2016	Identify the most promising obesity prevention intervention characteristics associated with successful behavioural and/or anthropometric outcomes.	18/47 Children 2–6 years, early care and education centres	All study designs with pre- and post-evaluation using objective or validated measures 4 RCT, 4 cluster-RCT, 3 randomised cross-over trial, 6 pre-post design, 1 quasi-experimental trial	Tentative evidence that multi-component and multi-level ECS interventions with parental engagement are most likely to be effective. AN: Healthy eating and parental involvement correlated with favourable anthropometric outcomes. DI: Most studies showed at least one positive dietary effect. No correlations found between HE intervention strength (calculated by authors using own system) and HE outcomes, with or without parental engagement.	Comprehensive, multi-level Stronger interventions with parental engagement and environmental and policy components Research already-effective interventions Explore whether comprehensiveness is negatively associated with feasibility and fidelity if educator led
Ward, Bélanger et al., 2015	Identify if childcare educators’ practices are associated with pre-schoolers’ physical activity and eating behaviours. Assess the effectiveness of interventions that control educators’ practices or behaviours	5/15 Pre-schoolers, educators, childcare facilities	All types of quantitative studies, excluding multi-component interventions or studies focusing on more than educators. 1 cross-over RCT, 2 quasi-experimental, 2 pre-post design	Educators may play a positive role in promoting healthy eating behaviours in children. DI: Significant, positive changes in dietary intake, particularly fruit and vegetables. Increased intake and acceptance of new or healthy food/snacks (5/5).	Educators have a crucial role in promoting HE behaviours in children Involve peers as change agents for positive eating Reassess interventions in today’s changed environment, use diverse populations, use objective or validated measurements
Ward, Bélanger et al., 2016	Effectiveness of the relationship between pre-schoolers’ eating behaviours and physical activity, and those of their peers.	7/13 Children 2–5 years, childcare centres	All types of quantitative studies 1 RCT, 3 pre-post design, 3 non-RCT	All nutrition interventions reported peers may influence eating behaviours. Social influences particularly modelling was a strong determinant of individual’s food intake. Moderated by number of peers, age, gender, perceived personality of role models. DI: Significant increase in targeted foods (7/7).	Use peers as agents for positive eating behaviours
Wolfenden, Jones et al., 2016	Effectiveness of strategies improving the implementation of policies, practices or programmes by childcare services that promote child healthy eating, physical activity and/or obesity prevention.	8/10 Children up to 5–6 years, centre-based childcare	Any study with a parallel control group that compared any strategy to improve the implementation of a healthy eating policy, practice or programme to no intervention, ‘usual’ practice or an alternative strategy and Included baseline. 1 RCT, 3 cluster-RCT, 2 quasi-experimental trial, 1 randomised CCT, 1randomised parallel-group trial	No intervention improved the implementation of all policies and practices targeted by the implementation strategies relative to a comparison group. Most reported at least one favourable change to policies or practices (7/8). DI: Significant positive changes in types of foods provided and foods selected. Consumed significantly less energy, fat, saturated fat compared to control in one study. AN: Significant reduction in centre-level child adiposity compared to control in one study. No significant intervention effect in one study following menu changes.	Include institutional changes: policy, health promotion, education, staff training, curriculum Assess cost-effectiveness Use comprehensive theoretical frameworks to identify implementation barriers Further determine barriers to implementation with formative research
Zhou, Emerson et al., 2014	Efficacy of childhood obesity interventions in childcare settings on outcomes of dietary intake, physical activity, and adiposity.	13/15 Children up to 5–6 years, preschool/ formal childcare	Any interventions with controlled study design 12 RCT-Cluster, 1 cluster controlled	Interventions variably effective in improving adiposity and dietary behaviours	Include institutional changes: policies, age-appropriate health promoting education curricula, educators’ training Include cost-effectiveness studies Research improving nutrition environments and target diverse populations Use consistent outcome measures, validated or objective measurements Add sufficient follow-up time

Abbreviations: AN anthropometrics; CCT controlled clinical trial; BCS behavioural change strategies; BCT behavioural change theory; BMI body mass index; DI dietary intake; ECEC Early Childhood Education and Care; ECS Early Childhood Service; HE healthy eating; PA physical activity; RCT randomised controlled trial; SBT social behavioural theory; SCT social cognitive theory; SEM social ecological model.

**Table 2:** Summarised research and practice recommendations by review authors.

Research Recommendations	Author	Practice Recommendations	Author
Future research should build upon existing activities	[38,55]	ECS have potential as settings for effective nutrition promotion	[38,53,54,57,58,61]
Include cost-effectiveness	[38,56,61]	Underpin intervention design with effective social behavioural change theory (e.g., Social Ecological Model, Social Cognitive Theory)	[38,39,52,53,54,57,58]
Be driven by user involvement (educators, parents) and children’s views	[39,52,58]	Target intervention strategies at environmental-level and individual-level determinants. Successful outcomes are more likely with a multi-component, multi-level approach	[39,54,55,56,58,59]
Measure children’s dietary changes as well as environmental impact	[39,54,57]	Involve and engage parents in intervention strategies. Changes are more likely with high levels of parental engagement	[38,39,52,53,54,55]
Include formative research to (1) determine barriers to strategy implementation (2) identify implementation drivers and barriers to increase understanding of how interventions work	[56,58,61]	Build the capacity of educators, who also have a role in inviting parental participation	[38,52,53,56,59]
Have longer follow-up to allow for behavioural changes to have an impact and to measure longer-term outcomes	[39,56,58]	Build the capacity of parents and of children with educational, hands-on experiences	[39,52,53,54]
Include more high-quality RCT with larger sample sizes using validated measurements and tools.	[54,56,59,60]	Involve peers (children) as change agents for positive eating behaviours	[59,60]
Explore whether collaborative parental engagement effects change	[54,55]	Include institutional changes; policies, age-appropriate education curricula, educators’ training	[56,61]

Abbreviations: ECS early childhood services, RCT randomised control trial.

## Supplementary Materials

The following are available online at http://www.mdpi.com/2072-6643/10/3/293/s1, Table S1: Record of search strategies, Table S2: Critical appraisal results for the included reviews using 11 critical appraisal criteria (The Johanna Briggs Institute 2014), Table S3: Characteristics of included systematic reviews (using JBI Data Extraction Form for Systematic Reviews and Research Syntheses, Aromataris et al., 2015, The Johanna Briggs Institute 2014), Table S4: Summary of the evidence from selected reviews using the JBI data extraction checklist (The Johanna Briggs Institute 2014), Figure S1: PRISMA flowchart of the selection process for systematic reviews.
