# Supplementary material for: Characteristics of Effective Interventions Promoting Healthy Eating for Pre-Schoolers in Childcare Settings: An Umbrella Review
Source: Nutrients. 2018 Mar 1;10(3):293. doi: 10.3390/nu10030293 (PMC5872711; doi:10.3390/nu10030293)
Supplement: Supplementary file 1 [file nutrients-10-00293-s001.zip › Table S4_Summary of the evidence from selected reviews.pdf]

Table S4: Summary of the evidence from selected reviews using the JBI data extraction checklist (The Johanna Briggs Institute 2014)

Author/Year of publication	Outcomes assessed	Total number of primary studies included/<br><br>Significance/direction of included diet-related studies	Summary of findings of included studies	Underpinning theories stated in the reviews	Summary of research limitations and recommendations	Summary of practice recommendations of included studies
Bell & Golley 2015	*Primary*<br>Children's dietary intake<br>*Secondary*<br>Centre environment<br>Centre food provision/availability<br>Parental food provision<br>Child knowledge/attitudes/preferences<br>Parent/knowledge<br>Staff knowledge/attitudes/behaviours	25 studies<br><br>Nearly all in the direction of nutritional improvement. Significance was $p<0.05$	*Primary*<br>Studies effect on children's dietary intake (8 out of 11 studies).Increases in fruit and<br>vegetable consumption [5 studies] and decreases in 'discretionary' food or saturated fat intake [3 studies]<br>*Secondary*<br>Studies with improvements in centres' nutrition environments including policy, nutrition best practices (6/6), nutritional quality of centres' menus (3/3), parental food provision (3/4 studies with three programs), child knowledge/attitudes/ preferences (2/2), staff knowledge/attitudes/ behaviours (4/4).<br><br>22/25 studies effective. Three studies were not: a pilot multi-component program, a program with a Latino population possibly because of low literacy skills, an intervention were the shape of snacks was changed	9 interventions reported using BCT; SCT n=7, SEM n=3 and SLT n=3.<br><br>More than 9 theories are listed as some studies used more than one<br><br>16/25 no theory reported	Evaluation of dietary intake beyond fruit and vegetables, and nutrient intake beyond fat and fibre was limited<br>- outcome assessment beyond evaluation of the nutrition environment also limited<br>- limitations include lack of comparison groups, poor intervention fidelity, selection and measurement bias, poor management of potential confounding in data analysis. However all RCTs or derivative and quality assessed.<br><br>Recommendations<br>- Underpin intervention design with theory<br>-Strongest evidence relates to the nutrition environment of children in care. Less robust evidence exists on the potential to influence children's dietary intake. Recommend evaluating the effect of changes to the nutrition environment on children's dietary intake.	Support the proposition that ECS have good potential as avenues for effective nutrition promotion.<br>- Environmental interventions can achieve improvements in determinants of children's dietary intake.<br>- Intervention development should continue as a priority to inform policy and practice.<br>- Future intervention development needs to carefully consider the behavioural targets, modifiable determinants and utilise age-appropriate and effective behaviour change theory
Hesketh & Campbell 2010	Child anthropometrics, diet, physical activity, or sedentary behaviour	3 studies<br><br>Effects favoured nutritional intervention groups,	1/3 showed lower BMI increases at 1 and 2 years follow up<br>Identical program with	Not reported	-Most conducted in the USA<br>- Failure to report cost-effectiveness data<br>- Lack of reporting of	Include parental component and build knowledge and skills of carers and parents

		significance at $p<0.05$	mainly Latino children no changes post intervention, or at 1 and 2 year F/U<br>2/2 studies significant decrease in total serum cholesterol but no impact on height to weight ratio<br>*Diet outcomes*<br>- 2/2 studies significant decrease in saturated fat and total fat in snacks, and corresponding reduction in intake		theoretical base of interventions<br><br>Recommendations<br>- Future research should build on and extend existing research activities	- Interventions which showed evidence of success were designed to impact not only on knowledge but also on skills and competencies suggesting a social behavioural theory underpinning
Ling & Wen 2016	Child anthropometry<br>- BMI, BMI-P, BMI z-score, percent fat, skin-folds, waist circumference.<br>Specific outcomes for diet not listed but included in this review as examined diet-related intervention characteristics	13 studies<br><br>Effects favoured intervention groups significance at $p<0.05$in some studies	6/13 studies effected measures of BMI<br>-1/6 sig, effect BMI, BMI-P, percent fat<br>-1/6 sig. effect compared to control in underprivileged areas<br>-1/6 sig. effect in BMI, BMI-P at 12 month follow-up but not post-intervention<br>-1/6 sig. effect at 12 mth, 24 mth F/U but post-intervention not reported<br>-1/6 sig. effect on BMI-z, waist circumference<br>-1/6 sig. effect percent fat, skin-folds, waist circumference but not BMI<br><br>12/13 studies included nutrition and a PA component. All of the studies which effected anthropometrics included both nutrition and PA components (n=6). Studies with PA only or nutrition only (n=1) had no significant effect.<br><br>From the studies which	SEM (n=2), SCT and Zajonic's exposure effect (n=1), SDT (n=1), SDT and HBM (n=1), HBM and competence motivational theory (n=1) GST (n=1)<br><br>6/13 no theory reported	Recommendations<br>Future research should examine the effects of demographics (gender, ethnicity, SES, parental education, marital and employment status) on intervention effects<br>- Meta-analysis required to explore moderator effects, publication bias and small-study effects<br>- Cost-effectiveness data should be reported<br>-Providing preschool teachers with a health promotion opportunity to enhance their health knowledge, increase healthy behaviours, and reduce stress can increase program fidelity and quality	-Including a health-promoting component for preschool teachers in an intervention for preschool children may be warranted in future studies<br>- Incorporating SCT-based strategies in future interventions may be a fruitful approach to prevent overweight/obesity in preschool children:<br>(a) providing behavioural training to increase skill development<br>(b) emphasizing feelings of mastery<br>(c) setting short- and long-term goals<br>(d) increasing self-efficacy and self-regulation through individualized positive feedback<br>(e) role-modelling or providing opportunities for observational learning<br>- Teaching preschool children and their families about the benefits of

			influenced weight status, success was associated with: consistent messages through educational material across home and childcare; capacity building of parents; parents encouraged children to drink water; parental satisfaction and participation<br><br>10/13 had active parental involvement. Lack of parental involvement may account for limited success in all studies<br><br>11of 16 studies (including 3 studies examining PA only) were educator delivered. May affect program fidelity as only 5 programs effective.			healthy eating and physical activity and targeting both nutrition and physical activity is recommended in future intervention work<br>-Future prevention interventions in preschool children should :<br>-target both parents and children through interactive education and behavioural therapy with parents<br>-use age-appropriate interactive education and hands-on experiences with a focus on physical activity and nutrition with children<br>(although intervention effects were less than optimal)
Mikkelsen et al 2014	Anthropometrics<br>-BMI, z-scores for height and wt, weight to height measurements, serum cholesterol, skin-folds, prevalence obesity and o/w<br>Dietary<br>-Food consumption patterns, food preferences, willingness to try foods, Knowledge and attitude	26 studies<br><br>Most in the direction of nutritional improvement and significance at $p<0.05$	Healthy eating interventions increased fruit and veg consumption and nutrition related knowledge.<br>*Single exposure interventions*<br>- no studies had an effect on vegetable intake<br>- some effect for fruit intake<br>*Educational interventions*<br>- 1/11 lower BMI and BMI-P in intervention group at follow up<br>- 1 increase in fruit and vegetable consumption (5 others showed increase but not significant)<br>- 2 increased nutrition-related knowledge<br>- 2 increased identification of fruits and vegetables<br>*Multicomponent interventions*	6 used SCT or SLT, 2 used Piaget's developmental theory, 1 listed theory of multiple intelligences and 1 Zajonc's exposure theory<br><br>16/26 no theory	Recommendations<br>Longer follow up in studies required<br>- outcomes should include intermediate measures such as knowledge and consumption, not just BMI to measure effectiveness<br>- development of innovative data collection methods capturing whether children are able to express what they like to eat and food related knowledge is needed<br>-include process evaluation beyond revising educational materials and monitoring compliance. Need to focus on implementation drivers and barriers to increase understanding of what makes an intervention work	Single exposure strategy insufficient to increase vegetable consumption, educational component also required<br>- the more comprehensive the intervention strategy, the more likely the intervention will be successful<br>- interventions should be targeted towards disadvantaged groups through targeting relevant centres<br>- target consumption of healthy foods and increasing knowledge of healthy eating<br>- interventions more likely to be successful when taking actions on several

			- 6/7 increase in fruit and vegetable intake<br>- 1 decrease in relative risk of high serum cholesterol<br>- 1/1 increase in familiarity with novel foods<br>No significant effect on anthropometrics<br>Multi-component programs most effective			levels into account<br>- Evidence that ECEC interventions reduce inequalities in health as positive results with fruit and veg
Morris et al 2015	Anthropometric<br>- BMI, BMI z-score<br>- percentage body fat<br>- waist circumference<br>- % body fat<br>- prevalence of overweight/obesity<br>Dietary intake<br>-fruit and vegetable consumption, consumption of EDNp foods, consumption of water<br>-<br>- % calories from fat<br>- nutrient content in meals<br>Nutrition knowledge and attitudes<br>- parents<br>- children<br>Environment<br>- menu changes	12 studies<br><br>Direction of change for dietary results reported for 7 of 12 studies. Others not reported.	Positive weight changes in 6 studies<br>-6 reduction in overall or subgroup BMI<br>- 1 reduction in incidence of overweight<br>- 2 no changes in anthropometry despite change in parental and child knowledge and attitudes and child unhealthy-diet behaviours<br>- 1 no change in anthropometric or dietary outcomes<br>Secondary outcome relating to HE seen in all included studies<br>-significant improvement in fruit and vegetable and/or improvements in ' reduction of EDNP foods' and increased drinking of water reported for 7 studies. Outcomes not reported for other 5 studies where it was also examined.	3 SEM, 2 SCT, one each of: self-determination theory, HBM, motivational theory, not specified theories of early childhood development, Bandura social learning theory, Zarjonic exposure effect, TTM<br><br>5/15 no theory	High attrition was a problem<br>- Requiring parental attendance reduced compliance<br>- Parental confounders not controlled or adjusted for<br>- Self/parental report of outcomes a limitation<br>- However all RCTs or derivative and quality assessed.<br><br>Recommendations<br>Small amount of parental engagement around the curriculum (only 2/13) highlights significant area for future research in collaborative parental involvement<br>Future interventions must plan, implement and evaluate any parental intervention conducted with an ECEC service.	Interventions should include communication with parents on classroom activities and content and include a better understanding of collaborative parental engagement<br>- Capacity building of parents, educators and communities contributes to positive BMI outcomes<br>- Interventions should adequately plan and examine ways to increase parental satisfaction and therefore engagement in interventions<br>- ECEC educators have a role in inviting parental participation
Nixon et al 2012	Child anthropometry<br>- weight, BMI, BMI z-scores, weight or overweight/obese classification status, skin-fold measurements, or waist circumference<br>Dietary	4 studies<br><br>Effects favoured all diet-related intervention groups, significance at $p<0.05$	Child anthropometry<br>- 2/4 showed weight changes<br>Dietary<br>- 4/4 showed improved dietary behaviours	SLT (n=1), Self-determination Theory + SLT (n=1), HBM (n=1), no theory listed for one study	Recommendations<br>Future research should focus on interventions that impact the built environment<br>- Should ensure evidence	Behavioural change strategies are key, rather than the use of specific behavioural models<br>- high parental

	- fruit and vegetable intake, intake of water and beverages, snacking behaviour, and nutrient intake		Overall, interventions that combined high levels of parental involvement and interactive school-based learning plus targeted dietary change and included long term follow-up were most effective<br><br>Rated level of parental involvement as high, medium, low.. All 4/4 diet-related studies had a significant outcome and high parental involvement.		base is driven by user involvement and children's views	involvement and programmes targeting both dietary and PA changes<br>- focus on developing children's (and parents') perceived competence at making dietary and physical changes, by implementing one or more of:<br>1. Developing skills and behavioural capability<br>2. Developing self-efficacy<br>3. Educating parents and children (in classroom-based and/or practical sessions) about the benefits of healthful dietary and PA behaviours<br>4. Modelling healthful eating and PA.<br>Need to explore changing environment to support diet rather than behavioural approaches aimed at the individual.<br>Need to ensure evidence base is driven by user involvement
Sisson et al 2016	Diet<br>- food intake at home/care, beverage intake at home/care,<br>Environment<br>- menus, policies, staff/parent behaviours	45 studies<br><br>Majority in the direction of nutritional improvement and significance was p<0.05	Diet<br>- 39/45 showed an effect in at least one nutrition outcome (87% desired effect)<br><br>-Child care centre policies and practices can be improved by intervention, demonstrating the environment is amendable to change, although environment-level only interventions had less impact on child health behaviour outcomes than those that	SCT (n=13), SEM (n=9), other theory (n=9),<br><br>No theory (n=14)<br><br>25/29 diet related studies based on behavioural theory had desired dietary behaviour change	-Lack of consistent outcome measures<br><br>Recommendations<br>-Use RCT and behavioural theory to influence obesity outcomes<br>-To sustain changes in children, need to emphasise parental involvement and include behave change strategy (SEM, SCT)<br>-Consider changes to control groups too (no changes with intervention but undesirable changes with control)	Multi-level approach (child, environment), multi-component re weight (diet and PA) recommended<br>-focus on child care environment, including technical support and training<br>-focus on child including educational component<br>-include parental involvement as correlated with favourable changes<br>-inclusion of behavioural change strategy eg SCT or

			specifically included child-level interventions		-Measure child's dietary changes as well as environmental changes for impact	SEM
Ward, S et al 2015	Dietary intake<br>- fruit and vegetable consumption<br>- healthy food consumption<br>-willingness to try or consume new foods	5 studies<br><br>All in the direction of nutritional improvement (increases in fruit and vegetable consumption), and significance was p<0.05	5/5 reported positive changes in dietary intake<br>- increased intake of new foods<br>- increased intake of fruits and vegetables<br>- increased intake and acceptance of healthy food/snacks	Not reported.<br>Theory of observational learning suggests children's behaviour shaped by watching educators.	Lack of representativeness of the target population<br>- Low response rates<br>- Lack of reporting of randomisation and blinding<br>- Tools for primary outcome measures not described as valid, or were not reported in the study or in a separate study.<br>- 4/5 studies published before 2000<br>- Most published in USA-Inadequate description of sample<br><br>Recommendations<br>More/better quality research required to provide recommendations for practice<br>-reassess interventions in today's changed environment and with more reliable measures<br>-use larger and more diverse populations<br>-explore effect of children preparing food or informal conversations during about food<br>-assess how peers who are picky eaters influence children's intake<br>-ensure representativeness, increase length of follow-up, use valid reliable and objective measurement tools, ensure reliability and validity of tools reported	Weak evidence that educator practices positively influence preschoolers' eating behaviours<br>- Educators can play a role in promoting healthy eating behaviours in children in childcare<br><br>Despite weak evidence that educators' positively influence children's eating behaviours, educators have a crucial education role and have a role promoting new guidelines<br><br>Involving peers as change agents for positive eating is recommended
Ward S, et al	Dietary intake/choice	7 studies	Social influences particularly	Not reported	:	Weak evidence that peers

2016	-fruit and vegetable consumption<br>- healthy food consumption<br>-<br>Food preference/acceptance<br>- ‘<br>-willingness to try or consume new foods	All in the direction of nutritional improvement, and significance was p<0.05	modelling was a strong determinant of individual’s food intake<br>-Moderated by age, gender, perceived personality of role models<br>- in 2 studies choice of non-preferred food increased<br>- Increased intake of target foods<br>- More bites of new foods<br>- Increased acceptance of new foods<br>-increases in fruit and vegetable consumption	Concept of social facilitation and observational learning theories suggested as relevant	- Convenience sampling and lack of report of response rates suggests poor representation of target population<br>- Low response rates<br>- Lack of information on validity and reliability of outcome assessment tools<br>- Missing numbers of withdrawals and dropouts<br><br>Recommendations<br>Recommend high-quality RCTs with larger sample sizes using reliable and validated tools	influence pre-schoolers’ eating behaviours.<br>- Future obesity prevention interventions aiming at reaching a large number of children should consider involving peers as agents for positive eating behaviours in pre-schoolers.
Ward D, et al 2016	Child anthropometry<br>-BMI, body fat, waist circumference, waist-to-height ratio, weight, MUAC<br>Dietary intake<br>-<br>-fruit and vegetable consumption<br>- food and beverage consumption	18 studies<br><br>Majority in the direction of nutritional improvement, and significance was p<0.05	Dietary intake<br>- 13/18 showed at least one positive effect<br>- 5/18 no change.<br>-some didn’t achieve results in every variable measured but only within some specific food groups or nutrients such as fruit, vegetable, sugar.<br>- all four intervention measures (ie intervention strength, parental engagement, study quality, intervention elements) negatively correlated for healthy eating intervention strength and dietary intake outcomes<br>-possibly due to small under-powered numbers, outliers or multi-component interventions and complex policy and environmental changes over time may be difficult to implement.<br>-made more difficult if delivered by child care staff.	Not reported for 13 studies<br>Generally consistent with ecological models of behavior (SEM) and recommendations from authoritative groups favouring multi-level comprehensive interventions (	Recommendations<br>Extent and quality of intervention implementation should be addressed, including the role of intervention complexity.<br>- Anomalous findings regarding intervention strength and behavioural outcomes should be examined further.<br>- Feasibility and effectiveness of single-behaviour versus combined physical activity and healthy eating interventions requires more focused study.<br>- Future research should use and improve the developed intervention strength scoring system<br>- It may be more productive to evaluate improved implementation<br>of already-effective interventions than to study novel combinations of intervention strategies	- Stronger interventions, with parent engagement and environmental and policy components tend to be positively related to anthropometric outcomes<br>- Comprehensive, multi-level obesity prevention interventions in ECE are recommended

			-Authors hypothesize that comprehensiveness may be negatively associated with feasibility or fidelity of implementation<br><br>HE and parental involvement correlated with favourable anthropometric outcomes<br><br>However study design not correlated with HE outcomes Also no correlations between HE intervention strength (calculated by authors using own system) and HE outcomes, with or without parental engagement		New hypothesis; more complex the intervention the less likely to be feasible or have fidelity, particularly if educator led	
Wolfenden et al 2016	Weight status<br>- BMI z-score<br>- weight to height ratio<br>Childcare staff knowledge, skills, attitudes<br>Dietary intake<br>-fruit and vegetable consumption<br>- food and beverage consumption<br>-energy, macronutrient intake, intake of key micronutrients<br>Types of foods provided<br>-	8 studies<br><br>Significance was p<0.05 in the same direction for majority of diet-related interventions<br><br>.<br><br>.	Weight status<br>- 1 reduced centre-level BMI z-score in intervention group, no difference in proportion of children in BMI groupings<br>- 1 no change in weight height ratio<br>-1 providing intensive intervention support in NAPSACC programs resulted in decreased child body mass index (BMI) z-score<br><br>No intervention improved the implementation of all policies and practices targeted by the implementation strategies relative to a comparison group.<br><br>Dietary intake<br>-1 intervention decreased energy, fat, saturated fat, fat as a percentage of energy and saturated fat as a	3/8 were theoretically based: 2 components of social cognitive theory against a social-ecologic framework, 1 x practice change and capacity building<br><br>5/8 not reported	- Lack of blinding of participants and personnel but this is difficult in rel-world conditions<br>- Use of self-assessment outcome measures a limitation<br>- Lack of prospective registration of trials a limitation<br>- Lack of consideration of power in sampling and small sample sizes a limitation<br><br>- Cost of implementation not assessed<br>-However all RCTs or derivative, quality assessed as part of Cochrane review<br><br>Recommendation<br>- include cost-effectiveness studies<br>- Formative work required to determine barriers to implementation of programs<br>-include more high quality	Uncertain whether the strategies tested improved the implementation of policies, practices or programmes that promote child healthy eating, physical activity and/or obesity prevention.<br><br>Highlights dearth of guidance available for policy-makers and practioners interested in supporting HE implementation strategies in centre-based childcare<br><br>Institutional changes recommended; policy, health promotion, educational, curriculum, training staff<br><br>Use of comprehensive theoretical frameworks to consider a broad range of

			percentage of energy at 6 months<br>- 1 intervention decreased saturated fat, fat as a percentage of energy and saturated fat as a percentage of energy at 18 months<br>- 1 intervention increased intake of iron and magnesium at 18 months<br><br>Types of food provided<br>-studies, positive changes in types of food provided<br>-studies positive changes in types of foods selected.<br>-studies positive changes in types of food served to children as part of a multi-component intervention or staff wellness program (n=2) relative to control services.<br>-studies effects also reported for energy and fat following one-day workshop to cooks by dietitian and within group changes to all types of food provided following two training support strategies (n=1)<br>-multi-component strategies targeting the foods provided and including implementation support through training likely to be effective n=3<br><br>Other<br>- 1 no difference in staff knowledge or attitude<br><br>-<br>- - Evaluation reported improvements in HE and PA policies but not in HE or PA practices (providing more		RCT with larger sample sizes using validated measurements and tools	implementation barriers when designing implementation support strategies

			support for strength of policies)			
Zhou et al 2012	*Primary*<br>Adiposity, measured as BMI, BMI z score, waist circumference, skin-fold thickness, or percent overweight or obese.<br>*Secondary*<br>Dietary and physical activity behaviours,	13 studies<br><br>Intervention group vs. control group, significance was p<0.05for the majority of studies	Childhood obesity prevention interventions were variably effective in improving adiposity, dietary behaviours<br><br>11/13 diet-related studies had significant. changes to adiposity and/or diet<br><br>Studies with studies changes in adiposity had diet **and** PA component (single component e.g. diet or PA only not studies. for changes in adiposity)<br>7/7 studies with sig changes to wt status included dietary-component, but 4 didn't record/measure diet outcomes<br><br>6/13 diet-related studies reported improvement in dietary intake in intervention groups versus control groups, including lower percentage of calories from saturated fat, higher intake of fruit and vegetables, fewer unhealthy lunch items, and increased frequency of eating breakfast.<br>Interventions that affected the environment (n=3 studies) were potentially sustainable by initiating institutional changes	Social cognitive theory (social learning theory) n=3 Zajonc's exposure effect n=1 health belief model n=1 competence motivational theory n=1 reinforced learning theory=1 self determination theory n=1<br><br>5 no theory stated	-Limitations include short intervention duration and short follow-up time period limiting sufficient intervention exposure and/or sufficient follow-up time to detect changes in adiposity beyond any intermediate behavioural changes.<br>-large variety of subjective measures for the secondary outcomes of diet, self-reports and 24-hr dietary recall<br>-sub-group analysis or studies with very diverse groups culturally and SES. cautions use of generalisability and transferability to different pops.<br><br>Recommendations<br>More research on interventions to improve the nutrition environment in preschool settings<br>-further test multi-strategy approaches<br>-use consistent outcome measures<br>- apply more narrow eligibility criteria for meta-analysis as more RCTs of childhood obesity interventions in childcare settings become available<br>- focus on targeting interventions to meet the needs of children from diverse cultural and socioeconomic backgrounds<br>- include cost-effectiveness	Interventions that incorporate institutional changes are important for sustainability, such as policies, age appropriate health promotion education curricula, and professional training of preschool staff.

					analysis of the intervention strategies and outcomes	

Abbreviations: BCT behaviour change theory, BMI body mass index, BMI-P body mass index percentile ECS Early Childhood Service, FFQ food frequency questionnaire, F/U follow-up GST generalised system theory HBM health belief model, HE healthy eating, MUAC mid-upper arm circumference NAPSACC nutrition and physical activity self-assessment for child care , OW overweight, SCT social cognitive theory, SEM socio-ecological model, SLT social learning theory, TTM transtheoretical model of change, Wt weight
