# Supplementary material for: Characteristics of Effective Interventions Promoting Healthy Eating for Pre-Schoolers in Childcare Settings: An Umbrella Review
Source: Nutrients. 2018 Mar 1;10(3):293. doi: 10.3390/nu10030293 (PMC5872711; doi:10.3390/nu10030293)
Supplement: Supplementary file 1 [file nutrients-10-00293-s001.zip › Table S1_Record of search strategies.pdf]

Effectiveness of interventions, strategies and practices on healthy eating in children 2-5 years in childcare: a systematic review of reviews

Table S1: Record of search strategies

All searches run 17/2/17

Database	Platform	Number of retrieved citations
Medline 1946-present, includes Epub Ahead of Print; In-Process & Other Non-Indexed Citations; Ovid MEDLINE(R) Daily	Ovid	239
Emcare, 1995 to 2017 week 06	Ovid	303
PsycINFO, 1806 to February Week 1 2017	Ovid	30
Embase, 1974 to 2017 February 16	Ovid	649
CINAHL	EBSCOhost	11
Joanna Briggs Institute EBP Database, Feb 08, 2017-	Ovid	0
Cochrane Database of Systematic Reviews, Cochrane Database of Systematic Reviews: Issue 2 of 12, February 2017	Wiley	3
Health Technology Assessment Database: Issue 4 of 4, October 2016	Wiley	1
ERIC	ProQuest	7
Scopus		249
Web of Science Core Collection		162
**Total before duplicates removed**		**1654**
**Total after duplicates removed**		**912?**

Ovid MEDLINE(R) Epub Ahead of Print, In-Process & Other Non-Indexed Citations, Ovid MEDLINE(R) Daily and Ovid MEDLINE(R) 1946 to Present

#	Searches	Results
1	Child Care/	5284
2	Child Day Care Centers/	4649
3	(Day care or Daycare or Childcare or "in child care" or "long day child child" or Occasional care or Out-of-home care or Nurser* or ((Preschool* or Pre-school* or Kindergarten* or Child care or Early child* or Early learning or Early education* or early years or home-based or centre-based or center based) adj3 (care or educat* or center* or centre* or setting* or attend* or provider* or work* or service* or home* or enrol* or environment* or policy or policies or facility or facilities))).tw,kw.	36274
4	or/1-3	40821
5	Child, Preschool/	821652
6	(Child* or (Age* adj1 ("2-5" or "3-5")) or "Under 5" or "Under 5s" or Toddler* or Preschooler* or Pre-schooler* or pre-primary).tw,kw.	1196867
7	or/5-6	1593221
8	exp Diet/ or Child nutrition sciences/ or Child Nutritional Physiological Phenomena/ or Food services/ or Food habits/ or Food preferences/ or Menu planning/ or Cooking/ or Fruit/ or Vegetables/ or Food/ or Energy intake/ or Meals/ or Lunch/ or Snacks/ or Feeding behavior/ or Eating/ or Drinking/ or Nutritional requirements/ or Nutritional status/ or Recommended daily allowances/ or "Fruit and Vegetable Juices"/ or Milk/	481612
9	(Diet* or Nutrition* or Eat or Eater* or Eating or Feed or Feeding or Food* or Drink* or Beverage* or Juice* or Milk* or Fruit* or Vegetables or Menu* or Lunch* or Breakfast* or snack* or Morning tea* or Afternoon tea* or ((Energy or calori* or kilojou* or nutrient*) adj1 (intake or consum*)) or ((portion* or serving or plate*) adj1 (size* or wast*)) or cook* or cater*).tw,kw.	1315314
10	or/8-9	1436846
11	4 and 7 and 10	3761
12	(meta analys* or metaanalys*).mp,pt. or review*.ti,pt. or (search* or MEDLINE or systematic review or synthesis).tw.	3196110
13	11 and 12	332
14	(infan* not child*).ti.	169275
15	13 not 14	314
16	(case reports or comment or editorial or letter or news).pt.	3357292
17	15 not 16	307
18	limit 17 to yr="2000 -Current"	239

Ovid Emcare 1995 to 2017 week 06

#	Searches	Results
1	child care/ or kindergarten/ or nursery/	19350
2	day care/	3477
3	(Day care or Daycare or "day child care" or Childcare or "in child care" or "long day child care" or Occasional care or Out-of-home care or Nurser* or ((Preschool* or Pre-school* or Kindergarten* or Child care or Early child* or Early learning or Early education* or early years or home-based or centre-based or center based) adj3 (care or educat* or center* or centre* or setting* or attend* or provider* or work* or service* or home* or enrol* or environment* or policy or policies or facility or facilities))).tw,kw.	18404
4	or/1-3	32522
5	preschool child/	69625
6	(Child* or (Age* adj1 ("2-5" or "3-5")) or "Under 5" or "Under 5s" or Toddler* or Preschooler* or Pre-schooler* or pre-primary).tw,kw.	387495
7	or/5-6	409350
8	exp diet/ or nutritional science/ or child nutrition/ or catering service/ or feeding behavior/ or eating habit/ or food preference/ or portion size/ or cooking/ or fruit/ or fruit juice/ or "fruit and vegetable juice"/ or vegetable juice/ or food/ or caloric intake/ or meal/ or fast food/ or feeding behavior/ or drinking/ or eating/ or nutritional requirement/ or nutritional status/ or milk/	172475
9	(Diet* or Nutrition* or Eat or Eater* or Eating or Feed or Feeding or Food* or Drink* or Beverage* or Juice* or Milk* or Fruit* or Vegetables or Menu* or Lunch* or Breakfast* or snack* or Morning tea* or Afternoon tea* or ((Energy or calori* or kilojou* or nutrient*) adj1 (intake or consum*)) or ((portion* or serving or plate*) adj1 (size* or wast*)) or cook* or cater*).tw,kw.	326887
10	or/8-9	347638
11	4 and 7 and 10	2653
12	(meta analys* or metaanalys*).mp,pt. or review.ti,pt. or (search* or MEDLINE or synthesis).ab. or systematic review.tw,sh.	709924
13	11 and 12	341
14	(infan* not child*).ti.	37959
15	13 not 14	328
16	(editorial or letter or note).pt.	691896
17	15 not 16	326
18	limit 17 to yr="2000 -Current"	303

PsycINFO 1806 to February Week 2 2017

#	Searches	Results
1	child care/ or child day care/ or preschool students/ or nursery school students/ or kindergarten students/	22585
2	(Day care or Daycare or Childcare or "day child care" or "in child care" or "long day child care" or Occasional care or Out-of-home care or Nurser* or ((Preschool* or Pre-school* or Kindergarten* or Child care or Early child* or Early learning or Early education* or early years or home-based or centre-based or center based) adj3 (care or educat* or center* or centre* or setting* or attend* or provider* or work* or service* or home* or enrol* or environment* or policy or policies or facility or facilities))).ti,ab,id.	35883
3	or/1-2	49333
4	(Child* or (Age* adj1 ("2-5" or "3-5")) or "Under 5" or "Under 5s" or Toddler* or Preschooler* or Pre-schooler* or pre-primary).ti,ab,id.	622000
5	preschool age 2 5 yrs.ag.	115538
6	or/4-5	637379
7	diets/ or eating behavior/ or fast food/ or food preferences/ or nutrition/ or food/ or fast food/ or food intake/ or food preparation/	47095
8	(Diet* or Nutrition* or Eat or Eater* or Eating or Feed or Feeding or Food* or Drink* or Beverage* or Juice* or Milk* or Fruit* or Vegetables or Menu* or Lunch* or Breakfast* or snack* or Morning tea* or Afternoon tea* or ((Energy or calori* or kilojou* or nutrient*) adj1 (intake or consum*)) or ((portion* or serving or plate*) adj1 (size* or wast*)) or cook* or cater*).ti,ab,id.	212956
9	or/7-8	214881
10	3 and 6 and 9	1873
11	(systematic review or meta analysis or metasynthesis).md,mp. or (search or medline or synthesis).ti,ab.	115007
12	10 and 11	33
13	(infan* not child*).ti.	29128
14	12 not 13	32
15	(column* or comment* or editorial or letter).dt.	179335
16	14 not 15	32

Embase 1974 to 2017 February 16

#	Searches	Results
1	child care/ or kindergarten/ or nursery/ or day care/	53995
2	(Day care or Daycare or "day child care" or Childcare or "in child	43368

	care" or Occasional care or Out-of-home care or Nurser* or ((Preschool* or Pre-school* or Kindergarten* or Child care or Early child* or Early learning or Early education* or early years or home-based or centre-based or center based) adj3 (care or educat* or center* or centre* or setting* or attend* or provider* or work* or service* or home* or enrol* or environment* or policy or policies or facility or facilities))).tw,kw.	
3	or/1-2	80284
4	preschool child/	541354
5	(Child* or (Age* adj1 ("2-5" or "3-5")) or "Under 5" or "Under 5s" or Toddler* or Preschooler* or Pre-schooler* or pre-primary).tw,kw.	1478970
6	or/4-5	1731472
7	exp diet/ or nutritional science/ or child nutrition/ or catering service/ or feeding behavior/ or eating habit/ or food preference/ or portion size/ or cooking/ or fruit/ or fruit juice/ or "fruit and vegetable juice"/ or vegetable juice/ or food/ or caloric intake/ or meal/ or fast food/ or feeding behavior/ or drinking/ or eating/ or nutritional requirement/ or nutritional status/ or milk/	764626
8	(Diet* or Nutrition* or Eat or Eater* or Eating or Feed or Feeding or Food* or Drink* or Beverage* or Juice* or Milk* or Fruit* or Vegetables or Menu* or Lunch* or Breakfast* or snack* or Morning tea* or Afternoon tea* or ((Energy or calori* or kilojou* or nutrient*) adj1 (intake or consum*)) or ((portion* or serving or plate*) adj1 (size* or wast*)) or cook* or cater*).tw,kw.	1616195
9	or/7-8	1731079
10	3 and 6 and 9	6326
11	(meta analys* or metaanalys*).mp,pt. or review.ti,pt. or (search* or MEDLINE or synthesis).ab. or systematic review.tw,sh.	3335034
12	10 and 11	797
13	(infan* not child*).ti.	176689
14	12 not 13	749
15	(editorial or letter or note).pt.	2183151
16	14 not 15	748

CINAHL (EBSCOhost)

#	Query	Limiters/Expanders	Results
S1	(MH "Child Day Care") OR (MH "Schools, Nursery")	Search modes - Boolean/Phrase	2,248
S2	TI (("Day care" OR Daycare OR "day child care" OR Childcare OR "in child care" OR	Search modes - Boolean/Phrase	12,779

	"long day child care" OR “Occasional care” OR “Out-of-home care” OR Nurser* OR ((Preschool* OR “Pre-school*” OR Kindergarten* OR “Child care” OR “Early child*” OR “Early learning” OR “Early education*” OR “early years” OR “home-based” OR “centre-based” OR “center based”) N2 (care OR educat* OR center* OR centre* OR setting* OR attend* OR provider* OR work* OR service* OR home* OR enrol* OR environment* OR policy OR policies OR facility OR facilities)))) OR AB ((“Day care” OR Daycare OR Childcare OR "in child care" OR “Occasional care” OR “Out-of-home care” OR Nurser* OR ((Preschool* OR “Pre-school*” OR Kindergarten* OR “Child care” OR “Early child*” OR “Early learning” OR “Early education*” OR “early years” OR “home-based” OR “centre-based” OR “center based”) N2 (care OR educat* OR center* OR centre* OR setting* OR attend* OR provider* OR work* OR service* OR home* OR enrol* OR environment* OR policy OR policies OR facility OR facilities))))		
S3	S1 OR S2	Search modes - Boolean/Phrase	13,684
S4	TI ((Child* OR (Age* N0 ("2-5" OR "3-5")) OR "Under 5" OR "Under 5s" OR Toddler* OR Preschooler* OR “Pre-schooler*” OR “pre-primary”)) OR AB ((Child* OR (Age* N0 ("2-5" OR "3-5")) OR "Under 5" OR "Under 5s" OR Toddler* OR Preschooler* OR “Pre-schooler*” OR “pre-primary”))	Search modes - Boolean/Phrase	209,646
S5	(MH "Child, Preschool")	Search modes - Boolean/Phrase	102,931
S6	S4 OR S5	Search modes - Boolean/Phrase	250,454
S7	TI ((Diet* OR Nutrition* OR Eat OR Eater* OR Eating OR Feed OR Feeding OR Food* OR Drink* OR Beverage* OR Juice* OR Milk* OR Fruit* OR Vegetables OR Menu* OR Lunch* OR Breakfast* OR snack* OR “Morning tea*” OR “Afternoon tea*” OR ((Energy OR calori* OR kilojou* OR	Search modes - Boolean/Phrase	35,927

	nutrient*) N0 (intake OR consum*)) OR ((portion* OR serving OR plate*) N0 (size* OR wast*)) OR cook* OR cater*)) AND AB ((Diet* OR Nutrition* OR Eat OR Eater* OR Eating OR Feed OR Feeding OR Food* OR Drink* OR Beverage* OR Juice* OR Milk* OR Fruit* OR Vegetables OR Menu* OR Lunch* OR Breakfast* OR snack* OR “Morning tea*” OR “Afternoon tea*” OR ((Energy OR calori* OR kilojou* OR nutrient*) N0 (intake OR consum*)) OR ((portion* OR serving OR plate*) N0 (size* OR wast*)) OR cook* OR cater*))		
S8	(MH "Child Nutritional Physiology") OR (MH "Diet+") OR (MH "Child Nutrition") OR (MH "Food Services") OR (MH "Menu Planning") OR (MH "Food Preferences") OR (MH "Eating Behavior") OR (MH "Food Habits") OR (MH "Meal Preparation") OR (MH "Cooking") OR (MH "Fruit") OR (MH "Vegetables") OR (MH "Meals") OR (MH "Breakfast") OR (MH "Lunch") OR (MH "Snacks") OR (MH "Eating") OR (MH "Drinking Behavior") OR (MH "Nutritional Requirements") OR (MH "Dietary Reference Intakes") OR (MH "Fruit Juices") OR (MH "Milk")	Search modes - Boolean/Phrase	85,253
S9	S7 OR S8	Search modes - Boolean/Phrase	104,956
S10	S3 AND S6 AND S9	Search modes - Boolean/Phrase	625
S11	(MH "Systematic Review") OR (MH "Meta Analysis")	Search modes - Boolean/Phrase	39,187
S12	PT (Meta Analysis OR Meta Synthesis OR Systematic Review) OR TI (meta analys* OR metaanalys* OR "systematic review") OR AB (meta analys* OR metaanalys* OR "systematic review")	Limiters - Published Date: 20000101-20171231; Publication Type: Meta Analysis, Meta Synthesis, Systematic Review<br>Search modes - Boolean/Phrase	40,646
S13	S11 OR S12	Search modes - Boolean/Phrase	54,758
S14	S10 AND S13	Search modes -	11

		Boolean/Phrase	

Joanna Briggs Institute EBP Database - Current to February 08, 2017

#	Searches	Results
1	(Day care or Daycare or Childcare or "in child care" or "long day child child" or Occasional care or Out-of-home care or Nurser* or ((Preschool* or Pre-school* or Kindergarten* or Child care or Early child* or Early learning or Early education* or early years or home-based or centre-based or center based) adj3 (care or educat* or center* or centre* or setting* or attend* or provider* or work* or service* or home* or enrol* or environment* or policy or policies or facility or facilities))).ti,hw,sh.	21
2	(Child* or (Age* adj1 ("2-5" or "3-5")) or "Under 5" or "Under 5s" or Toddler* or Preschooler* or Pre-schooler* or pre-primary).ti,hw,sh.	337
3	(Diet* or Nutrition* or Eat or Eater* or Eating or Feed or Feeding or Food* or Drink* or Beverage* or Juice* or Milk* or Fruit* or Vegetables or Menu* or Lunch* or Breakfast* or snack* or Morning tea* or Afternoon tea* or ((Energy or calori* or kilojou* or nutrient*) adj1 (intake or consum*)) or ((portion* or serving or plate*) adj1 (size* or wast*)) or cook* or cater*).ti,hw,sh.	289
4	((Day care or Daycare or Childcare or "in child care" or "long day child child" or Occasional care or Out-of-home care or Nurser* or ((Preschool* or Pre-school* or Kindergarten* or Child care or Early child* or Early learning or Early education* or early years or home-based or centre-based or center based) adj3 (care or educat* or center* or centre* or setting* or attend* or provider* or work* or service* or home* or enrol* or environment* or policy or policies or facility or facilities))) and (Child* or (Age* adj1 ("2-5" or "3-5")) or "Under 5" or "Under 5s" or Toddler* or Preschooler* or Pre-schooler* or pre-primary) and (Diet* or Nutrition* or Eat or Eater* or Eating or Feed or Feeding or Food* or Drink* or Beverage* or Juice* or Milk* or Fruit* or Vegetables or Menu* or Lunch* or Breakfast* or snack* or Morning tea* or Afternoon tea* or ((Energy or calori* or kilojou* or nutrient*) adj1 (intake or consum*)) or ((portion* or serving or plate*) adj1 (size* or wast*)) or cook* or cater*)).ti,hw,sh.	0
5	limit 4 to systematic reviews	0

Web of Science Core Collection

N=162

((“Day care” OR Daycare OR Childcare OR "in child care" OR “long day child care” OR “Occasional care” OR “Out-of-home care” OR Nurser* OR ((Preschool* OR

“Pre-school*” OR Kindergarten* OR “Child care” OR “Early child*” OR “Early learning” OR “Early education*” OR “early years” OR “home-based” OR “centre-based” OR “center based”) NEAR/2 (care OR educat* OR center* OR centre* OR setting* OR attend* OR provider* OR work* OR service* OR home* OR enrol* OR environment* OR policy OR policies OR facility OR facilities))) AND (Child* OR (Age* NEAR/0 ("2-5" OR "3-5")) OR "Under 5" OR "Under 5s" OR Toddler* OR Preschooler* OR “Pre-schooler*” OR “pre-primary”) AND ((Diet* OR Nutrition* OR Eat OR Eater* OR Eating OR Feed OR Feeding OR Food* OR Drink* OR Beverage* OR Juice* OR Milk* OR Fruit* OR Vegetables OR Menu* OR Lunch* OR Breakfast* OR snack* OR Morning tea* OR Afternoon tea* OR ((Energy OR calori* OR kilojou* OR nutrient*) NEAR/0 (intake OR consum*)) OR ((portion* OR serving or plate*) NEAR/0 (size* OR wast*)) OR cook* OR cater*)))

Refined by: PUBLICATION YEARS: (2016 OR 2003 OR 2015 OR 2014 OR 2013 OR 2012 OR 2002 OR 2010 OR 2011 OR 2009 OR 2008 OR 2007 OR 2006 OR 2005 OR 2017 OR 2004 OR 2000 OR 2001) AND **DOCUMENT TYPES: (REVIEW)**
Timespan: All years. Indexes: SCI-EXPANDED, SSCI, A&HCI, CPCI-S, CPCI-SSH, ESCI, CCR-EXPANDED, IC.

ERIC (Educational Resources Information Center)

N=7

((“Day care” OR Daycare OR Childcare OR "in child care" OR “day child care” OR “Occasional care” OR “Out-of-home care” OR Nurser* OR ((Preschool* OR “Pre-school*” OR Kindergarten* OR “Child care” OR “Early child*” OR “Early learning” OR “Early education*” OR “early years” OR “home-based” OR “centre-based” OR “center based”) NEAR/2 (care OR educat* OR center* OR centre* OR setting* OR attend* OR provider* OR work* OR service* OR home* OR enrol* OR environment* OR policy OR policies OR facility OR facilities))) AND (Child* OR (Age* NEAR/0 ("2-5" OR "3-5")) OR "Under 5" OR "Under 5s" OR Toddler* OR Preschooler* OR “Pre-schooler*” OR “pre-primary”) AND ((Diet* OR Nutrition* OR Eat OR Eater* OR Eating OR Feed OR Feeding OR Food* OR Drink* OR Beverage* OR Juice* OR Milk* OR Fruit* OR Vegetables OR Menu* OR Lunch* OR Breakfast* OR snack* OR “Morning tea*” OR “Afternoon tea*” OR ((Energy OR calori* OR kilojou* OR nutrient*) NEAR/0 (intake OR consum*)) OR ((portion* OR serving or plate*) NEAR/0 (size* OR wast*)) OR cook* OR cater*)))

Scopus

n=249

(((TITLE-ABS-KEY ("Day care" OR daycare OR childcare OR "in child care" OR "day child care" OR "Occasional care" OR "Out-of-home care" OR "long day child care" OR nurser*)) OR (TITLE-ABS-

KEY (((preschool* OR "Pre-school*" OR kindergarten* OR "Child care" OR "Early child*" OR "Early learning" OR "Early education*" OR "early years" OR "home-based" OR "centre-based" OR "center based") near/2 (care OR educat* OR center* OR centre* OR setting* OR attend* OR provider* OR work* OR service* OR home* OR enrol* OR environment* OR policy OR policies OR facility OR facilities))))) AND (TITLE-ABS-KEY (child* OR ((age OR aged OR ages) W/0 ("2-5" OR "3-5")) OR "Under 5" OR "Under 5s" OR toddler* OR preschooler* OR "Pre-schooler*" OR "pre-primary"))) AND ((TITLE-ABS-KEY (diet* OR nutrition* OR eat OR eater* OR eating OR feed OR feeding OR food* OR drink* OR beverage* OR juice* OR milk*)) OR (TITLE-ABS-KEY (fruit* OR vegetables OR menu* OR lunch* OR breakfast* OR snack* OR "Morning tea*" OR "Afternoon tea*")) OR (TITLE-ABS-KEY (((energy OR calori* OR kilojou* OR nutrient*) W/0 (intake OR consum*)))) OR (TITLE-ABS-KEY (((portion* OR serving OR plate*) near/0 (size* OR wast*)))) OR (TITLE-ABS-KEY (((portion* OR serving OR plate*) W/0 (size* OR wast*)))) OR (TITLE-ABS-KEY (cook* OR cater*))) AND (LIMIT-TO (PUBYEAR , 2017) OR LIMIT-TO (PUBYEAR , 2016) OR LIMIT-TO (PUBYEAR , 2015) OR LIMIT-TO (PUBYEAR , 2014) OR LIMIT-TO (PUBYEAR , 2013) OR LIMIT-TO (PUBYEAR , 2012) OR LIMIT-TO (PUBYEAR , 2011) OR LIMIT-TO (PUBYEAR , 2010) OR LIMIT-TO (PUBYEAR , 2009) OR LIMIT-TO (PUBYEAR , 2008) OR LIMIT-TO (PUBYEAR , 2007) OR LIMIT-TO (PUBYEAR , 2006) OR LIMIT-TO (PUBYEAR , 2005) OR LIMIT-TO (PUBYEAR , 2004) OR LIMIT-TO (PUBYEAR , 2003) OR LIMIT-TO (PUBYEAR , 2002) OR LIMIT-TO (PUBYEAR , 2001) OR LIMIT-TO (PUBYEAR , 2000)) AND (LIMIT-TO (DOCTYPE , "re"))
