# Supplementary material for: Characteristics of Effective Interventions Promoting Healthy Eating for Pre-Schoolers in Childcare Settings: An Umbrella Review
Source: Nutrients. 2018 Mar 1;10(3):293. doi: 10.3390/nu10030293 (PMC5872711; doi:10.3390/nu10030293)
Supplement: Supplementary file 1 [file nutrients-10-00293-s001.zip › Table S2. Quality Assessment of Selected Reviews_Umbrella Review_manuscript.pdf]

Table S2: Critical appraisal results for the included reviews using 11 critical appraisal criteria (The Johanna Briggs Institute 2014)

Quality Assessment Criteria	Is the review question clearly and explicitly stated	Were the inclusion criteria appropriate for the review question	Was the search strategy appropriate	Were the sources and resources used to search for studies adequate	Were the criteria for appraising studies appropriate	Was critical appraisal conducted by two or more reviewers independently	Were there methods to minimize errors in data extraction	Were the methods used to combine studies appropriate	Was the likelihood of publication bias assessed	Were recommendations for policy and/or Practice supported by the reported data	Were the specific directives for new research appropriate	Included /Excluded
Bell & Golley 2015	Y	Y	Y	Y	Y	N	Y	Y	Y	Y	Y	Included
Campbell & Hesketh 2007	Y	Y	U	U	N	U	U	Y	N	N	N	Excluded
Hesketh & Campbell 2010	Y	Y	N	Y	Y	Y	Y	Y	Y	Y	Y	Included
Larson et al 2011	Y	U	N	N	N	U	N	Y	N	Y	Y	Excluded
Ling et al 2016	Y	Y	Y	Y	Y	Y	Y	Y	Y	Y	Y	Included
Mikkelsen et al 2014	Y	Y	Y	Y	Y	Y	Y	Y	Y	Y	Y	Included
Morris et al 2015	Y	Y	Y	Y	Y	U	Y	Y	N	Y	Y	Included
Nixon et al 2012	Y	Y	Y	Y	Y	Y	U	Y	Y	Y	Y	Included
Sisson et al 2016	Y	Y	Y	Y	N	Y	Y	Y	Y	Y	Y	Included
Ward, D et l 2016	Y	Y	Y	Y	Y	Y	Y	Y	Y	Y	Y	Included
Ward S, et al 2015	Y	Y	Y	Y	Y	Y	Y	Y	Y	Y	Y	Included
Ward S, et al 2016	Y	Y	Y	Y	Y	Y	Y	Y	Y	Y	Y	Included
Wolfenden et al 2016	Y	Y	Y	Y	Y	Y	Y	Y	Y	Y	Y	Included
Zhou et al 2012	Y	Y	Y	Y	Y	Y	Y	Y	Y	Y	Y	Included

N, no, critical appraisal criterion was not met; N/A, criteria appraisal criterion was not applicable; U, unclear whether critical appraisal criterion was met; Y, yes, critical appraisal criterion was met.
