# Supplementary material for: Characteristics of Effective Interventions Promoting Healthy Eating for Pre-Schoolers in Childcare Settings: An Umbrella Review
Source: Nutrients. 2018 Mar 1;10(3):293. doi: 10.3390/nu10030293 (PMC5872711; doi:10.3390/nu10030293)
Supplement: Supplementary file 1 [file nutrients-10-00293-s001.zip › Table S3_ Characteristics of included systematic reviews.pdf]

Repository

Table S3: Characteristics of included systematic reviews (using JBI Data Extraction Form for Systematic Reviews and Research Syntheses, Aromataris et al 2015, The Johanna Briggs Institute 2014)

Author/Year of publication	Systematic review objectives	Participant characteristics/sample/setting	Description of interventions	Sources searched	Year range of included studies	Number of included primary studies /Total number of diet-related studies in review (Total number of studies in review)	Types of studies/number and types of study design of included studies	Country of origin of included studies	Appraisal rating of included studies/Appraisal instrument used
Bell & Golley 2015	*Primary*<br>Evaluate the effectiveness of nutrition promotion interventions on children's dietary intake<br>*Secondary*<br>Evaluate environmental and individual factors; centre's nutrition policies, centre's practices, food provided, knowledge and attitudes of educators, parents, children	Children 0-5 years, providers and staff or parents of children in formal childcare care<br><br>Children n=28-8950 (total 18998)<br>Educators/directors/ cooks n=9-87 (total 385)<br>Staff n=30-496 (total 994)<br>Centres n=1-229 (total 575)	Any intervention that included a nutrition component targeting staff (e.g. training), children (e.g. nutrition curriculum) or parents/ caregivers (e.g. education sessions) that aimed to influence children's nutritional intake.	CINAHL, Medline (n=2) Reference lists and recent reviews	Up to June 2013	25/26 (excluded Bravo et al 2008 as FDC study)	Prospective studies with or without a comparison group, evaluating the effectiveness of an intervention of any duration, with outcomes measured at baseline and post intervention<br><br>9 cohort pre-post studies, 7 CCT, 1 pilot CCT, 1 cross-over study, 4 RCT, 1 cross-over cluster-RCT , 1 pilot cohort, 1 cross-over quasi-experimental design	USA (n=19), Australia (n=4), Germany (n=1) UK (n=1)	14 weak, 11 moderate quality
Hesketh & Campbell 2010	Evaluate effectiveness of interventions designed to prevent obesity, promote healthy eating and/or physical activity or reduce	Children 0-5 years<br>Family/home, group, primary care, pre-school/formal childcare and mixed settings	Any intervention designed to prevent obesity, promote healthy eating and/or	Academic Search Premier, Cumulative Index to	Jan 1995-Aug 2008	3/9 (23 studies in all settings)<br>9 in preschool or	Experimental studies<br>2 cluster- RCT, 1 CCT	Not reported but mostly in the USA. All diet-related studies in USA	2 moderate, 1 strong quality

	sedentary behaviours	Children n=1,810<br>Centres- not reported	physical activity or reduce sedentary behaviour	Nursing and Allied Health Literature, Cochrane Central Register of Controlled Trials, Communication, Global Health, Health Source: Nursing/Academic, Medline, Psycharticles, PsychINFO, Psychology, and Behavioral Sciences (n=10)<br>Reference lists<br>Contacted researchers with emerging studies		childcare, 3 of these diet related<br><br>Included studies: Fitzgibbon et al 2005, Fitzgibbon et al 2006, Williams et al 2004			
Ling & Wen 2016	*Primary*<br>Examine effects of prevention and management interventions on overweight/ obesity *Secondary*<br>Explore factors that may influence intervention effects	Formal childcare/preschool, community and home settings<br>Children aged 2–5 years<br><br>295 centres (range 4 to 79), 7,805 (range 101 to 1663) children	Any intervention that aimed to improve behaviours, including screen time, sedentary activity, physical activity, diet, and/or sleep	PubMed, CINAHL, EMBASE, PsycINFO, ERIC, and Cochrane library<br>n=6<br>Reference lists of reviews	Up to Feb 2015	13/19 (26 studies in all settings)<br>Nutrition/PA x 12 studies<br>Nutrition only x 1<br><br>Excluded studies which were PA only or sedentary behaviour only: Annesi et al 2013a, 2013b, Bonvin et al 2013, De Bock 2013, Dennison et al 2004, Reilly	13 Cluster RCTs<br>Included studies with a sample size > 30	USA (n = 6), Switzerland (n = 1), Germany (n = 1), Israel (n = 2), Belgium (n = 1), France (n=1)<br>Australia (n = 1)	1 study low risk of bias (Burgi et al 2012), others insufficient info for evaluation

						et al 2006.			
Mikkelsen et al 2014	Analyse the effectiveness of different strategies in relation to their influence on children's food choice at an early age, provide recommendations for future interventions	3 to 6 year-olds, educators – NOT parents<br>Preschools (13), kindergartens (10) and day care facilities (3)<br><br>16-6102 children<br>26 centres	Any healthy eating intervention attempting to prevent obesity with a focus on diet, nutrition, food, eating or meals in day care facilities	PubMed, Scopus, Web of Science and CINAHL (n=4)<br>Reference lists	1980-2014	26<br>8 single interventions<br>11 educational<br>7 multi-component	Intervention studies with baseline and follow-up measurements<br><br>11 RCTs, 9 quasi RCTs, 1 cross-over, 2 pre-post test design, 3 cluster -RCT	North America (n=17), South America (n=1), Asia (n=5), European context (n=3)	4 weak,<br>9 moderate, 10 strong,<br>3 very strong quality
Morris et al 2015	(1) How have parents been incorporated into childhood obesity interventions conducted in ECEC settings and to what extent, if any, does their involvement impact the outcomes of the intervention?<br>(2) What are the methodological limitations of ECEC childhood obesity prevention interventions that have included a parental component?<br>(3) What recommendations can be made for future research?	Parents of children in ECEC settings<br><br>22,267 children plus DeCoen's et al 2012 not reported (range 289-12000), 275 centres plus those from 4 unspecified studies<br>(range 7-64 centres)<br>Most trials recruited <20 centres (6/11)	Any interventions to prevent obesity or risk factors (diet, PA) with a parental component. Single setting interventions excluded.	Academic Source Complete, CINAHL, Global Health, ERIC, Health Source, Medline and PsychInfo (n=7)<br>Reference lists searched	2000-2014	12/15 studies Excluded Story et al 2012 (schools), Reilly et al 2006 (PA only), Dennison et al 2004 (sedentary behaviour only), De Bock et al 2013 (PA only). Included De Bock et al 2012.	Experimental studies<br><br>2 RCT, 6 cluster RCT, 3 quasi-experimental, 1 prospective cohort	USA (n=4), Australia (n=2) China (n=1), Belgium (n=1), Germany (n=2) Columbia (n=1) Switzerland (n=1)	6 fair, 7 good<br>No studies classified as excellent or poor
Nixon et al 2012	To identify the most effective behavioural models and behaviour change strategies, underpinning preschool and school-based interventions aimed at preventing obesity in 4–6-year-olds.	4-6 year olds<br>preschool- and school based<br><br>no sample sizes reported	Any preschool- or school based interventions for preventing obesity in 4–6-year-olds	MEDLINE, EMBASE, CINAHL, PsycINFO and The Cochrane Library (n=5)<br><br>Hand Searching of reviews and reference lists	1995-2010	4/9 (12 studies including schools)<br><br>4 studies diet-related (5 studies preschoolers but PA only. Four studies	RCT, Non-RCT With 'before and after' measures in the same children<br>Plus follow-up periods of 6 months or longer<br><br>1 RCT, 3 cluster	Germany (n=1), Nth America (n=1), Asia (n=1), Australia (n=1)	1 strong,<br>3 moderate, quality

						diet-related but school-based)	RCT		
Sisson et al 2016	Identify interventions that target obesogenic behaviours in child care centres and<br>(1) Examine the duration, use of behavioural theory, and intervention targets, including the child care environment, teacher, parents, and children;<br>(2) Describe the intervention strategies and their effectiveness	3-to-5-year-old children<br>Child care settings<br><br>no sample sizes reported	Interventions designed to reduce obesity and improve obesogenic behaviours, including physical activity, diet, and screen time, at child care centres.	PubMed, PsychInfo, and Ovid (n=3)<br>Manual searches of personal records were also conducted, along with screening of previous review articles and reference lists of identified articles	Up to January 2016	44/45 (71 interventions including PA only)<br>Excluded Cespedes et al 2013 as in Colombia<br><br>22 RCTs, 19 quasi-experimental or pre-post design, 3 non-experimental	All experimental designs were eligible	Not stated	22 Level II, 19 Level III, 3 Level IV
Ward S, et al 2015	To identify<br>a) if childcare educators' practices predict or are associated with pre-schoolers' physical activity and eating behaviours in childcare centres<br>b) to assess the effectiveness of interventions that control educators' practices or behaviours in order to improve pre-schoolers' physical activity and eating behaviours	Pre-schoolers, educators<br>Childcare facilities<br><br>19-97 children, 1-19 childcare centres	Any interventions assessing the impact of childcare educators' practices or behaviours on children's physical activity or eating behaviours	PubMed, The Cochrane Library, Science Direct, SportDiscus, CINAHL and Wiley (n=6)<br><br>Reference lists and reviews	Up to July 2015	5/15<br><br>Only 5 studies assessed diet, rest PA only<br><br>2 quasi-experimental, 2 pre-post design, 1 cross-over RCT	All types of quantitative study designs but multi-component interventions, or those where the study results could not be explained solely by the educators' practices or behaviours were excluded	5/5 USA for diet-related<br><br>USA (n=14), Netherland (n=1)	3 low, 2 moderate quality
Ward S, et al 2016	To examine the relationship between preschoolers' eating behaviours and physical activity, and those of their peers	2 and 5 years of age<br><br>No specific setting defined, but mostly childcare centres<br><br>14-66 children (total 260	Quantitative studies examining the relationship between preschoolers' eating behaviours	Science Direct, PsychInfo, PubMed, Medline, ERIC, SportDiscus and CINAHL	Up to July 2015	7/13<br><br>3 non-randomised controlled trials, 3 pre-	All types of quantitative studies, including non-randomized and observational	USA (n=4), England (n= 1), Wales (n= 1), Brazil (n=1)	2 moderate, 5 low quality

		children in 6 single-centre studies). Sample not reported for one study.	and physical activity, and those of their peers<br><br>How preschoolers' eating behaviours and physical activity relate to their peers' behaviours	(n=7)<br>Reference lists and reviews		post design, 1 RCT	type as can provide impetus for future RCT		
Ward, D et al 2016	To identify the most promising obesity prevention intervention characteristics associated with successful behavioural and/or anthropometric outcomes<br>1. Is **intervention strength** related to successful behavioural and/or anthropometric outcomes?<br>2. Are interventions that incorporate **parent engagemen**t more effective than those that do not?<br>3. Can specific **intervention elements** be identified that relate to desired outcomes, including number of intervention strategies used, potential impact of the strategies, and frequency and duration of these strategies?<br>4. Is overall **study quality** related to successful behavioural and/or anthropometric outcomes?<br><br>Developed a coding strategy to assess intervention strength and allow for examination of several study questions.	Children ages 0–6 years (actually children aged 2-6 years), mostly low and middle SES<br>Early care and education centres<br><br>1-31 centres, 57-2062 children	Any obesity prevention interventions in centre-based ECE settings<br><br>What specific intervention characteristics and strategies contribute to intervention effectiveness for obesity prevention in centre-based child care.	PubMed, ERIC, and Web of Science (n=3)<br>Reference searching of reviews and articles	2010-2015	18/43 unique interventions, 26 had dietary-component but only 18 reported outcomes<br><br>6 Pre-post design, 4 cluster RCT, 4 RCT, 3 randomised cross-over trial, 1 quasi-experimental trial	All study designs, except case studies, were included if a pre- and post-evaluation was conducted and used an objective or validated measure	US (n=11), Australia (n=1), Germany (n=1), Switzerland (n=1), Chile (n=1), Belgium (n=1), Spain (n=1), and Turkey (n=1)	3 strong, 4 moderate, and 11 weak global rating
Wolfenden et al 2016	*Primary*<br>Examine the effectiveness	Children typically up to the age of 5-6 years, staff	Any strategy with	Cochrane Central	Up to August	8/10	Any study (randomised or	USA (n=5), Australia (n=2),	All studies had high risk of bias

	of strategies aimed at improving the implementation of policies, practices or programmes by childcare services that promote child healthy eating, physical activity and/or obesity prevention.<br>*Secondary*<br>1. describe the impact of such strategies on childcare service staff knowledge, skills or attitudes;<br>2. describe the cost or cost-effectiveness of such strategies;<br>3. describe any adverse effects of such strategies on childcare services, service staff or children	Centre-based childcare services (preschools, nurseries, long day-care services and kindergartens that cater for children prior to compulsory schooling).<br><br>Most trials recruited <20 centres<br>1053 centres participated across all trials<br>5/8 diet-related studies in childcare services in disadvantaged areas or serving disadvantaged. 3/8 SES not described	the primary intent of improving the implementation<br><br>of policies, practices or programmes in centre-based childcare services to promote healthy eating, physical activity or prevent unhealthy weight gain	Register of Controlled trials (CENTRAL), MEDLINE, MEDLINE In Process, EMBASE, PsycINFO, ERIC, CINAHL and SCOPUS (n=8)<br>Reference lists of included trials, hand searched two international implementation science journals<br>World Health Organization International Clinical Trials Registry Platform and ClinicalTrials.gov	2015	2 healthy eating, 6 healthy eating and physical activity<br><br>3 Cluster-randomised controlled-trial, 2 quasi-experimental trial, 1 randomised trial, 1 randomised controlled trial, 1randomised parallel-group trial	non-randomised) with a parallel control group that compared any strategy to improve the implementation of a healthy eating, physical activity or obesity prevention policy, practice or programme to no intervention, 'usual' practice or an alternative strategy. Included baseline.	Ireland (n=1)	for at least one domain
Zhou et al 2012	*Primary*<br>To assess the efficacy of childhood obesity interventions in childcare settings<br>on outcomes of dietary intake, physical activity, and adiposity,<br><br>*Secondary*<br>To identify gaps and limitations of the existing studies and recommend priorities for future research.	Children up to school age Childcare facilities for preschool aged children who are not old<br>enough to attend primary or elementary schools (childcare centers, preschools, daycares, nursery schools, and kindergartens if childcare)<br><br>In the 13/15 diet-related studies total participants at baseline n=5620 (range 101-2658 children), # centres not recorded	Any intervention aimed at childhood obesity prevention with a controlled study design	PubMed, Web of Science, Cochrane Library, ERIC (n=4)<br>Reviewed reference lists of included intervention studies and other relevant review articles	Jan 2000-Aug 2012	13/15 studies<br><br>12 RCTs-Cluster, 1 cluster controlled design for diet-related	Any interventions aimed at childhood obesity prevention with; controlled study design (randomized or nonrandomized), outcome measures included adiposity (e.g., body mass index)	United States (n=4), Israel (n=3), Australia (n=1), Germany (n=2), France (n=1), Switzerland (n=1), China (n=1)	All studies rated high for performance bias, most others for attrition bias, one for selection bias. All low risk of bias for reporting.

Abbreviations: CCT controlled clinical trial; ECEC Early Childhood Education and Care; FDC Family Day Care; PA physical activity; RCT randomised controlled trial
