# Supplementary figures and images for: Characteristics of Effective Interventions Promoting Healthy Eating for Pre-Schoolers in Childcare Settings: An Umbrella Review
Source: Nutrients. 2018 Mar 1;10(3):293. doi: 10.3390/nu10030293 (PMC5872711; doi:10.3390/nu10030293)

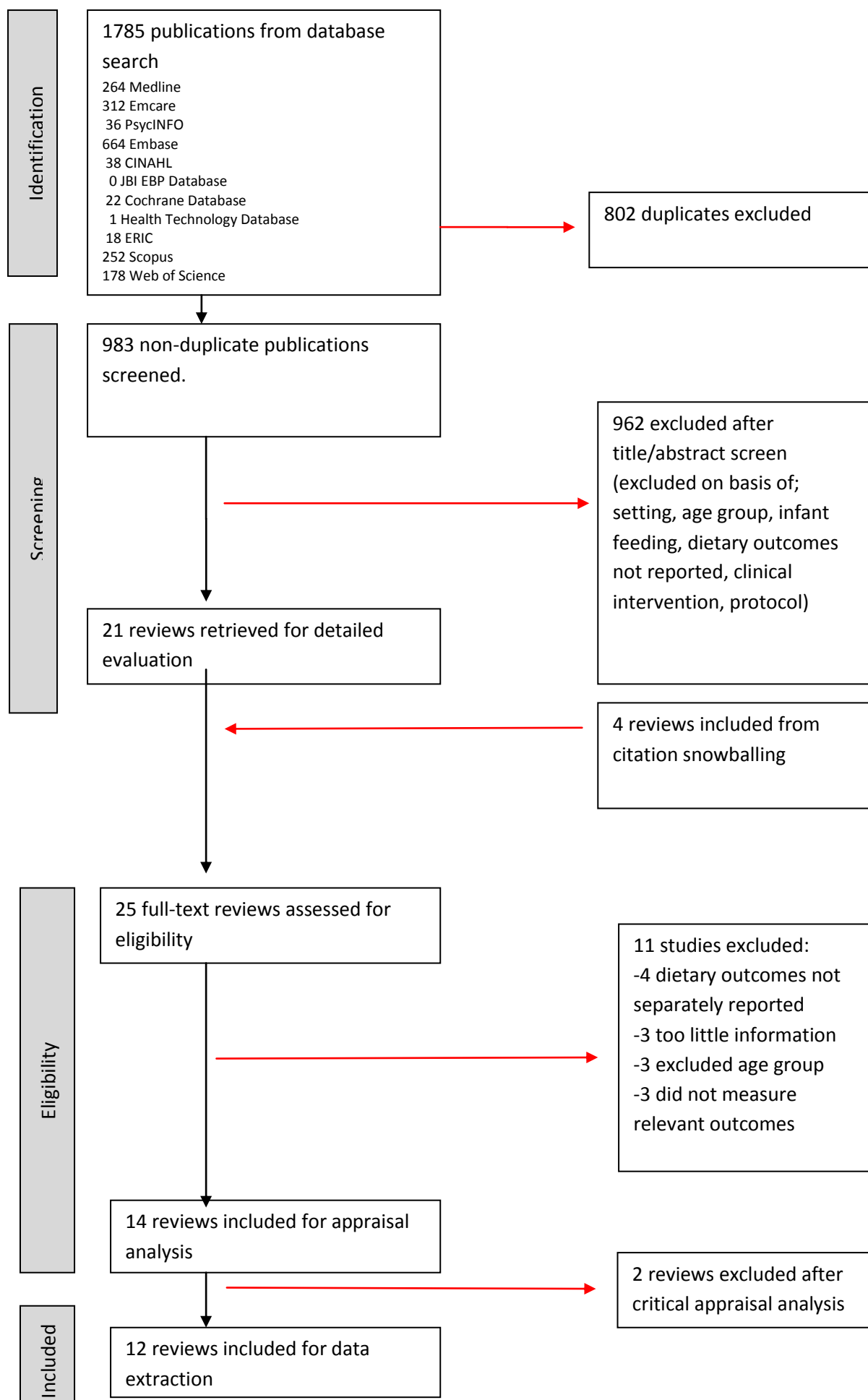

**Figure S1:** PRISMA flowchart of the selection process for systematic reviews

Supplement: Supplementary file 1 [file nutrients-10-00293-s001.zip › Figure S1_PRISMA flowchart of the selection process for systematic reviews.pdf]
